# Long-term Intake of Gluten and Cognitive Function Among US Women

**DOI:** 10.1001/jamanetworkopen.2021.13020

**Published:** 2021-05-21

**Authors:** Yiqing Wang, Benjamin Lebwohl, Raaj Mehta, Yin Cao, Peter H. R. Green, Francine Grodstein, Manol Jovani, Paul Lochhead, Olivia I. Okereke, Laura Sampson, Walter C. Willett, Qi Sun, Andrew T. Chan

**Affiliations:** 1 Clinical and Translational Epidemiology Unit, Massachusetts General Hospital, Harvard Medical School, Boston; 2 Division of Gastroenterology, Massachusetts General Hospital, Harvard Medical School, Boston; 3 Celiac Disease Center, Department of Medicine, Columbia University College of Physicians and Surgeons, New York, New York; 4 Department of Epidemiology, Mailman School of Public Health, Columbia University, New York, New York; 5 Division of Public Health Sciences, Department of Surgery, Washington University in St Louis, St Louis, Missouri; 6 Division of Gastroenterology, Department of Medicine, Washington University in St Louis, St Louis, Missouri; 7 Rush Alzheimer’s Disease Center, Rush University Medical Center, Chicago, Illinois; 8 Department of Internal Medicine, Rush Medical College, Chicago, Illinois; 9 Division of Gastroenterology and Hepatology, Johns Hopkins Hospital, Baltimore, Maryland; 10 Channing Division of Network Medicine, Department of Medicine, Brigham and Women’s Hospital and Harvard Medical School, Boston, Massachusetts; 11 Department of Psychiatry, Massachusetts General Hospital and Harvard Medical School, Boston; 12 Department of Epidemiology, Harvard T.H. Chan School of Public Health, Boston, Massachusetts; 13 Department of Nutrition, Harvard T. H. Chan School of Public Health, Boston, Massachusetts

## Abstract

**Importance:**

Gluten avoidance has been suggested as having a benefit to cognitive health among the general population, given the link between gluten and cognitive impairment in patients with celiac disease. However, data are lacking in individuals without celiac disease.

**Objective:**

To examine whether gluten intake is associated with cognitive function in women without celiac disease.

**Design, Setting, and Participants:**

This cohort study included US women who participated in the longitudinal, population-based Nurses’ Health Study II and had not previously or subsequently been diagnosed with celiac disease. Dietary data were collected from 1991 to 2015, and data on cognitive function were collected from 2014 to 2019. Data analysis was conducted from October 2020 to April 2021.

**Exposures:**

Energy-adjusted gluten intake, cumulatively averaged across questionnaire cycles prior to cognitive assessment.

**Main Outcomes and Measures:**

Three standardized cognitive scores assessed by the validated Cogstate Brief Battery: (1) psychomotor speed and attention score, (2) learning and working memory score, and (3) global cognition score. Higher scores indicated better performance.

**Results:**

The cohort included 13 494 women (mean [SD] age, 60.6 [4.6] years). The mean (SD) gluten intake was 6.3 (1.6) g/d. After controlling for demographic and lifestyle risk factors in linear regression, no significant differences in standardized cognitive scores (mean [SD], 0 [1]) by quintile of gluten intake were found across highest and lowest quintiles of gluten intake (psychomotor speed and attention: −0.02; 95% CI, −0.07 to 0.03; *P* for trend = .22; learning and working memory: 0.02; 95% CI, −0.03 to 0.07; *P* for trend = .30; global cognition: −0.002; 95% CI, −0.05 to 0.05; *P* for trend = .78). The null associations persisted after additional adjustment for major sources of dietary gluten (ie, refined grains or whole grains), comparing decile categories of gluten intake, using gluten intake updated at each previous questionnaire cycle, or modeling changes in gluten intake. Similarly, these associations were not materially altered in sensitivity analyses that excluded women who had reported cancer or dementia diagnosis or had not completed all dietary assessments.

**Conclusions and Relevance:**

In this study, long-term gluten intake was not associated with cognitive scores in middle-aged women without celiac disease. Our results do not support recommendations to restrict dietary gluten to maintain cognitive function in the absence of celiac disease or established gluten sensitivity.

## Key Points

**Question:**

Is dietary gluten intake associated with cognitive function in the general population?

**Findings:**

In this cohort study of 13 494 women at midlife without a celiac disease diagnosis, there was no statistical evidence of any association of long- or short-term gluten intake with cognitive function.

**Meaning:**

These findings suggest that in the absence of celiac disease, restriction of dietary gluten to maintain cognitive function is not warranted.

## Teaser

This cohort study examines whether gluten intake is associated with cognitive function in women without celiac disease.

## Introduction

Gluten is a protein present in wheat, barley, and rye.^1^ It is a ubiquitous component of Western diet, with an estimated intake of 5 to 20 g/d.^1^ In celiac disease, gluten triggers severe immune responses characterized by autoantibodies and villus atrophy.^2^ This immune reaction has been linked to multiple neuropsychiatric symptoms, including cognitive impairment, depression, and anxiety, as summarized in several reviews.^3,4,5^ A longitudinal study of 11 patients aged 22 to 39 years with celiac disease showed that following a gluten-free diet for a year resulted in mucosal healing and modest improvement in cognitive performance.^6^

Although gluten is not generally believed to cause harm to individuals without celiac disease, gluten-free diets have become increasingly popular in the United States. According to the National Health and Nutrition Examination Survey,^7^ the percentage of people without celiac disease avoiding gluten increased from 0.5% in 2009 to 1.7% in 2014, while the prevalence of celiac disease remained low during the same period (0.7%). In addition, 2 online surveys of more than 1000 US residents conducted in 2015 reported that 20% to 21% of consumers avoided dietary gluten,^8,9^ with a higher percentage in women (23%) than men (19%). Since the 2013 publication of *Grain Brain*,^10^ a New York Times best-selling book that has been translated into more than 30 languages, the assertion that gluten has a deleterious effect on cognitive health has become popularized among the lay public. However, there is a lack of epidemiological study demonstrating the association between gluten intake and cognitive function among individuals without celiac disease or nonceliac gluten sensitivity. Therefore, we aimed to investigate whether long-term gluten intake is associated with cognitive function in a cohort of women at midlife without celiac disease, using dietary data collected over 2 decades and cognitive scores assessed with validated tools.

## Methods

### Study Population

We used the Nurses’ Health Study II (NHS II), a nationwide prospective cohort of female nurses aged 25 to 42 years when enrolled in 1989.^11^ Lifestyle and health information was collected using biennial questionnaires. Dietary information had been collected every 4 years since 1991 using a validated food frequency questionnaire (FFQ).^12,13,14^ The return of mailed questionnaires implied informed consent. More than 90% of potential person-years were retained in follow-ups. Since 2014, 40 082 women with known email addresses and information on trauma exposure and posttraumatic stress disorder were invited to the Cognitive Function Study.^15^ Women who completed the cognitive assessment and provided plausible dietary information (600-3500 kcal/d) were eligible for the analysis. Participants who responded to this cognitive assessment were similar to the nonresponders in terms of age, body mass index (BMI; calculated as weight in kilograms divided by height in meters squared), alcohol intake, physical activity, and depressive symptoms.^15^ We excluded women who did not complete the full cognitive battery, had implausibly low cognitive scores that reflected likely technical errors, had previous diagnosis of stroke, or had previous or subsequent diagnosis of celiac disease, as these conditions may affect cognition and gluten intake. This study has been approved by the institutional review board of the Brigham and Women’s Hospital and followed the Strengthening the Reporting of Observational Studies in Epidemiology (STROBE) reporting guideline for cohort studies.

### Assessment of Gluten Intake

Our primary exposure was long-term gluten intake estimated every 4 years since 1991 using a 131-item semiquantitative FFQ (eFigure 1 in the Supplement), which has been validated by 1-week food diaries and biomarkers of diet,^13,14^ with a Spearman correlation coefficient of 0.58 for gluten and 0.60 for gluten-rich food.^12,16^ We identified gluten-containing foods, such as wheat, rye, and barley, based on year-specific ingredient lists from manufacturers or cookbooks. The quantity of gluten-containing ingredients in each food was calculated by multiplying the serving size with amounts of ingredients per serving. We then estimated gluten content by multiplying the protein proportions of these ingredients by 0.75, in line with previous studies.^17,18,19,20,21,22,23^ Next, we added up gluten contents in all foods to estimate total gluten intake in grams per day. To account for confounding by energy intake and potential measurement errors, we calculated energy-adjusted intake using residuals from the regression of absolute intake on total energy intake (ie, the residual method).^24^ We averaged gluten intake from 1991 to the last FFQ preceding the cognitive assessment to quantify long-term intake. We divided energy-adjusted gluten intake by quintiles (0.1 to <5.1, 5.1 to <5.9, 5.9 to <6.6, 6.6 to <7.6, and 7.6 to 18.3 g/d) to assess potential nonlinear associations and threshold of effect.

### Assessment of Cognitive Function

The outcomes of this study were 3 standardized cognitive scores measured by the validated, self-administered, online Cogstate Brief Battery that consisted of 4 tasks.^25,26,27^ This battery has been demonstrated to be a reliable assessment for mild cognitive impairment^28^ and has been found to have comparable performance in unsupervised and supervised settings.^29^ We calculated the score for psychomotor speed and attention by averaging task scores on Detection (DET), during which the participant pressed a key each time a card flipped over, and Identification (IDN), during which the participant pressed the yes or no key to indicate whether a red or black card flipped over. We calculated the score for learning and working memory by averaging task scores on One Card Learning (OCL), during which the participant responded yes or no to indicate whether a card had been shown before, and One Back (ONB), during which the participant responded yes or no to indicate whether the card was the same as the previous card. We averaged the scores on all 4 tasks to represent the global cognition score. As previously described,^30^ we calculated DET, IDN, and ONB scores by reversing the *z* normalized and log_10_ transforming mean response times for correct answers, and we calculated the OCL score by *z* normalizing the arcsine square-root transformed percentage of correct responses. We restandardized these 3 composite cognitive scores using *z* normalization. Therefore, a higher score indicated better cognitive performance, with each unit increase representing 1 SD higher than the mean score. Maruff et al^31^ have shown that these composite scores were sensitive in identifying cognitive impairment and had high test-retest reliability (*r* = 0.95) in 643 healthy adults and 151 adults with mild cognitive impairment or Alzheimer disease.

### Statistical Analysis

We used age- and multivariable-adjusted linear regression to examine the associations between gluten intake quintiles and each cognitive score. Consistent with prior analyses of data from our cohort,^22,23,32,33^ we tested linear trend across gluten categories by assigning the median quintile value to each quintile (4.45, 5.48, 6.23, 7.03, and 8.34 g/d) and testing it as a continuous variable. To help interpret the model results, we calculated age-equivalent differences in cognitive scores by dividing the regression coefficient of gluten by that of age obtained in the same model.

In our multivariable model constructed a priori, we included the following potential confounders as covariates: self-reported race (White or other racial/ethnic group), husband’s education as an indicator for socioeconomic status because nurses had similar education backgrounds (high school or below, college, graduate school, unmarried, or missing [n = 391]), family income (in 2010 US $),^34^ personal history of hypertension (yes or no),^35^ diabetes (yes or no), hypercholesterolemia (yes or no), myocardial infarction (yes or no),^36^ current depression status (yes, indicating diagnosis, treatment, and/or clinically relevant depressive symptoms according to the 10-item Centre for Epidemiological Studies Depression Scale [score ≥10], or no, indicating none of the previously listed options),^37,38^ BMI, smoking status (never, past, current, or missing [n = 12]),^39^ regular use of aspirin or nonsteroidal anti-inflammatory drugs (NSAIDs; yes or no), use of multivitamins (yes or no), physical activity (metabolic-equivalents [METs]/week),^40^ menopausal status and menopausal hormone use (premenopausal or postmenopausal with no use, past use, or current use of hormone therapy), total energy intake (kcal/d), alcohol intake (g/d), and the Alternative Healthy Eating Index 2010 (AHEI-10, excluding alcohol and whole grains).^33^ We acquired these variables from the most recent questionnaire prior to cognitive testing, except for dietary factors, BMI, and physical activity, which were averaged across previous questionnaires to capture long-term patterns and reduce variance.^32^ Race information was collected for the NHS II because these data could provide valuable information on differences in disease risk and risk factors by race and ethnicity and help reduce health disparities by race and ethnicity. The authors categorized the racial/ethnic groups as White and other racial/ethnic group because the study cohort was predominantly White individuals (96%). When missing values occurred, we imputed with median values for family income (n = 22), BMI (n = 5), and AHEI-10 (n = 8), and carried forward the last nonmissing responses for aspirin or NSAID use (n = 2).

We conducted several secondary analyses. First, because grains are major sources of gluten and are correlated with cognition,^41^ we additionally adjusted for refined grains (servings per day) and whole grains (servings per day) in the multivariable model. Then, we conducted stratified analyses by age (<65 vs ≥65 years), smoking (never vs ever), BMI (<25 vs ≥25), current depression status, and history of diabetes, hypertension, and hypercholesterolemia to investigate potential effect modification by testing the interaction of gluten and these factors. Next, given that it is possible that only extreme intake of gluten affects cognition, we compared cognitive scores by gluten intake deciles. Last, we modeled the continuous gluten variable in grams per day to test for linear associations. We also conducted several sensitivity analyses to assess the robustness of our findings. First, we additionally excluded participants who (1) had ever reported cancer diagnosis (n = 1761), (2) had every reported dementia diagnosis (n = 8), (3) or had not completed all dietary assessments preceding the cognitive assessment (n = 1848). Second, to assess temporal associations, we modeled gluten intake during each 4-year questionnaire cycle before the cognitive assessment (≤4, 4-8, 8-12, 12-16, 16-20, and 20-24 years), distant past (12-24 years), recent past (4-12 years), and per 1-SD change in mean gluten intake (mean [SD], −0.87 [1.98] g/d) from distant to recent past.

We used SAS version 9.4 (SAS Institute) to perform all analyses. All tests were 2 sided and had a significance level of *P* < .05. The eMethods in the Supplement provide more details on measurements and analysis.

## Results

Among 13 494 women (mean [SD] age, 60.6 [4.6] years; mean [SD] gluten intake, 6.3 [1.6] g/d) in the analytic cohort, the mean (SD) energy-adjusted gluten intake ranged from 4.3 (0.6) g/d in the lowest quintile to 8.6 (1.0) g/d in the highest quintile (Table 1; eFigure 2 in the Supplement). The mean age, BMI, physical activity, and diet quality were similar across gluten intake quintiles. Whereas participants in the lowest quintile tended to have the highest percentages of hypertension (1132 of 2700 [41.9%]), diabetes (236 [8.7%]), and hypercholesterolemia (1666 [61.7%]), participants in the highest quintile tended to have the highest percentages of depression (898 of 2699 [33.3%]) and never smoking (1908 [70.7%]).

**Table 1.** Age-Adjusted Characteristics of Women Participating in the Nurses’ Health Study II, According to Quintiles of Mean Energy-Adjusted Gluten Intake^a^

Characteristics	Participants by quintile of mean gluten intake, No. (%)				
	1, lowest (n = 2700)	2 (n = 2697)	3 (n = 2699)	4 (n = 2699)	5, highest (n = 2699)
Energy-adjusted gluten intake, mean (SD), g/d	4.3 (0.6)	5.5 (0.2)	6.2 (0.2)	7.0 (0.3)	8.6 (1.0)
Age at cognitive testing, mean (SD), y^b^	61.2 (4.5)	60.7 (4.6)	60.6 (4.6)	60.3 (4.5)	60.0 (4.7)
White individuals	2497 (92.5)	2603 (96.5)	2644 (98.0)	2627 (97.3)	2642 (97.9)
Husband’s educational attainment					
≤High school	422 (15.6)	443 (16.4)	419 (15.5)	407 (15.1)	377 (14.0)
College	1185 (43.9)	1185 (43.9)	1178 (43.6)	1117 (41.4)	1094 (40.5)
Graduate school	737 (27.3)	775 (28.7)	763 (28.3)	852 (31.6)	902 (33.4)
Unmarried or missing	356 (13.2)	294 (10.9)	339 (12.6)	323 (12.0)	326 (12.1)
Family income, mean (SD), in thousands of 2010 US $	82.1 (31.9)	82.1 (31.1)	83.7 (31.6)	84.6 (32.6)	85.6 (32.2)
BMI, mean (SD)	26.5 (5.7)	26.5 (5.5)	26.4 (5.6)	26.0 (5.2)	25.5 (5.4)
Currently diagnosed with depression or had clinically relevant depressive symptoms	784 (29.0)	849 (31.5)	875 (32.4)	871 (32.3)	898 (33.3)
History of hypertension	1132 (41.9)	1114 (41.3)	1046 (38.8)	965 (35.8)	900 (33.4)
History of diabetes	236 (8.7)	220 (8.2)	216 (8.0)	169 (6.3)	150 (5.6)
History of hypercholesterolemia	1666 (61.7)	1611 (59.7)	1620 (60.0)	1547 (57.3)	1434 (53.1)
History of myocardial infarction	33 (1.2)	46 (1.7)	35 (1.3)	31 (1.1)	30 (1.1)
Menopausal status and hormone use					
Premenopausal	260 (9.63)	331 (12.3)	324 (12.0)	341 (12.6)	378 (14.0)
Postmenopausal and never used hormones	1021 (37.8)	1079 (40.0)	1084 (40.2)	1148 (42.5)	1183 (43.8)
Postmenopausal and formerly used hormones	547 (32.3)	450 (31.0)	486 (29.8)	484 (26.9)	441 (25.8)
Postmenopausal and currently using hormones	872 (20.3)	837 (16.7)	805 (18.0)	726 (17.9)	697 (16.3)
Smoking status					
Never	1645 (60.9)	1785 (66.2)	1768 (65.5)	1826 (67.7)	1908 (70.7)
Past	929 (34.4)	819 (30.4)	856 (31.7)	803 (29.8)	730 (27.0)
Current	124 (4.6)	89 (3.3)	73 (2.7)	67 (2.5)	60 (2.2)
Missing	2 (0.1)	4 (0.1)	2 (0.1)	3 (0.1)	1 (0.04)
Multivitamin use	1536 (56.9)	1527 (56.6)	1531 (56.7)	1441 (53.4)	1408 (52.2)
Regular aspirin or NSAID use	678 (25.1)	679 (25.2)	671 (24.9)	654 (24.2)	630 (23.3)
Physical activity, mean (SD), METs/wk	24.5 (20.3)	22.2 (18.2)	22.9 (18.8)	23.1 (19.6)	22.7 (18.6)
Total calories, mean (SD), kcal/d	1795.0 (436.7)	1823.0 (424.6)	1821.3 (416.2)	1809.6 (438.9)	1744.1 (413.2)
Alcohol intake, mean (SD), g/d	5.9 (8.3)	5.5 (7.4)	5.4 (7.1)	5.3 (7.0)	4.6 (6.2)
AHEI-10 score, mean (SD)^c^	47.3 (9.6)	45.8 (8.7)	45.8 (8.4)	46.3 (8.4)	45.9 (8.2)
Refined grain intake, mean (SD), servings/d	0.9 (0.4)	1.1 (0.5)	1.1 (0.5)	1.3 (0.6)	1.4 (0.6)
Whole grain intake, mean (SD), servings/d	0.8 (0.5)	1.0 (0.5)	1.1 (0.6)	1.3 (0.7)	1.5 (0.9)

Abbreviations: AHEI-10, Alternative Healthy Eating Index 2010; BMI, body mass index (calculated as weight in kilograms divided by height in meters squared); MET, metabolic equivalent; NSAID, nonsteroid anti-inflammatory drug.

^a^ Values are adjusted for age (49-55, 56-60, 61-65, and 66-70 y). All variables were updated until the cognitive testing. Dietary intake, BMI, and physical activity were cumulatively averaged through 1991 to the most recent survey (2011 or 2015) before cognitive testing.

^b^ Value is not age adjusted.

^c^ The AHEI-10 score excluded whole grains and alcohol, so it included the following 9 components, each scored from 0 to 10, with higher scores indicating healthier diet and better adherence to the index: vegetables, fruits, sugar-sweetened beverages and fruit juice, nuts and legumes, red and/or processed meat, trans fat, long-chain (n3) fatty acids, poly-unsaturated fatty acids, and sodium.

When estimating age-adjusted mean differences in standardized cognitive scores across quintiles of gluten intake (Table 2), we found a slight increase in learning and working memory score between the highest and lowest quintiles (0.05; 95% CI, −0.004 to 0.10; *P *for trend = .04) and no statistically significant differences in psychomotor speed and attention score (−0.005; 95% CI, −0.06 to 0.05; *P *for trend = .55) and global cognition score (0.02; 95% CI, −0.03 to 0.07; *P *for trend = .50). After adjusting for various behavioral and health risk factors in the multivariable model, we observed no difference in psychomotor speed and attention score (−0.02; 95% CI, −0.07 to 0.03; *P *for trend = .22), learning and working memory score (0.02; 95% CI, −0.03 to 0.07; *P* for trend = .30), and global cognition score (−0.002; 95% CI, −0.05 to 0.05; *P* for trend = .78) across quintiles of gluten intake. These multivariable-adjusted estimates were equivalent to mean differences in psychomotor speed and attention, learning and working memory, and global cognition scores associated with 0.5 years, −0.67 years, and 0.05 years of aging, respectively.

**Table 2.** Differences in Standardized Cognitive Scores Associated With Quintiles of Gluten Intake Among 13 494 Women^a^

Model	Differences by quintiles of gluten intake, mean (95% CI)					
	1, lowest	2	3	4	5, highest	*P* value for trend^b^
**Psychomotor speed and attention**^c^						
Standardized score, mean (SD)	−0.03 (1.03)	0.01 (1.00)	0.02 (0.96)	−0.002 (1.02)	0.02 (0.99)	NA
Age-adjusted	0 [Reference]	0.01 (−0.04 to 0.07)	0.02 (−0.03 to 0.07)	−0.02 (−0.07 to 0.04)	−0.005 (−0.06 to 0.05)	.55
Multivariable-adjusted^d^	0 [Reference]	0.01 (−0.04 to 0.06)	0.01(−0.04 to 0.06)	−0.02 (−0.08 to 0.03)	−0.02 (−0.07 to 0.03)	.22
Multivariable-adjusted, with additional adjustment for refined grains^d^	0 [Reference]	0.01 (−0.04 to 0.07)	0.02 (−0.03 to 0.07)	−0.02 (−0.07 to 0.04)	−0.01 (−0.06 to 0.05)	.51
Multivariable-adjusted, with additional adjustment for whole grains^d^	0 [Reference]	0.01 (−0.04 to 0.07)	0.02 (−0.03 to 0.07)	−0.01 (−0.07 to 0.04)	−0.002 (−0.06 to 0.06)	.66
**Learning and working memory**^c^						
Standardized score, mean (SD)	−0.04 (0.98)	0.0001 (0.96)	0.07 (0.98)	0.05 (0.98)	0.05 (0.96)	NA
Age-adjusted	0 [Reference]	0.03 (−0.03 to 0.08)	0.09 (0.04 to 0.14)	0.06 (0.01 to 0.11)	0.05 (−0.004 to 0.10)	.04
Multivariable-adjusted^d^	0 [Reference]	0.01 (−0.04 to 0.06)	0.07 (0.02 to 0.13)	0.04 (−0.01 to 0.09)	0.02 (−0.03 to 0.07)	.30
Multivariable-adjusted, with additional adjustment for refined grains^d^	0 [Reference]	0.01 (−0.04 to 0.06)	0.07 (0.02 to 0.12)	0.04 (−0.02 to 0.09)	0.02 (−0.04 to 0.07)	.49
Multivariable-adjusted, with additional adjustment for whole grains^d^	0 [Reference]	0.01 (−0.04 to 0.07)	0.07 (0.02 to 0.13)	0.04 (−0.01 to 0.09)	0.02 (−0.03 to 0.08)	.33
**Global cognition**^c^						
Standardized score, mean (SD)	−0.05 (1.02)	−0.001 (0.98)	0.05 (0.97)	0.02 (1.02)	0.03 (0.97)	NA
Age-adjusted	0 [Reference]	0.02 (−0.03 to 0.08)	0.06 (0.01 to 0.11)	0.02 (−0.03 to 0.07)	0.02 (−0.03 to 0.07)	.50
Multivariable-adjusted^d^	0 [Reference]	0.01 (−0.04 to 0.07)	0.05 (−0.004 to 0.10)	0.01 (−0.05 to 0.06)	−0.002 (−0.05 to 0.05)	.78
Multivariable-adjusted, with additional adjustment for refined grains^d^	0 [Reference]	0.02 (−0.04 to 0.07)	0.05 (−0.002 to 0.10)	0.01 (−0.05 to 0.06)	−0.003 (−0.05 to 0.06)	.95
Multivariable-adjusted, with additional adjustment for whole grains^d^	0 [Reference]	0.02 (−0.04 to 0.07)	0.05 (0.001 to 0.11)	0.01 (−0.04 to 0.07)	0.01 (−0.05 to 0.07)	.82

Abbreviation: NA, not applicable.

^a^ Energy-adjusted gluten intake was cumulatively averaged from 1991 to the last questionnaire cycle preceding the cognitive assessment. To infer age-equivalent differences in standardized cognitive scores, parameter estimates for age in the same multivariable-adjusted models for gluten intake were −0.04 (95% CI, −0.04 to −0.03), −0.03 (95% CI, −0.04 to −0.03), and −0.04 (95% CI, −0.05 to −0.04) for psychomotor speed and attention score, learning and working memory score, and global cognition score, respectively.

^b^ *P* value for trend was calculated using the median value for each gluten intake quintile as a continuous variable.

^c^ Standardized scores for psychomotor speed and attention, learning and working memory, and global cognition were calculated by standardizing the mean standardized scores of the following Cogstate battery tasks: Detection and Identification, One Card Learning and One Back, and all 4 tasks, respectively. Higher scores indicate better performance, with 1 unit increase representing 1 SD higher than the mean.

^d^ Adjusted for age, race, body mass index, husband’s educational attainment, family income, history of diabetes, history of hypertension, history of hypercholesterolemia, history of myocardial infarction, current depression status, smoking, aspirin or nonsteroid anti-inflammatory drug use, multivitamin use, physical activity, menopausal status and hormone use, total energy intake, alcohol intake, and Alternative Healthy Eating Index score, excluding alcohol and whole grains.

After additionally adjusting for refined grains or whole grains (Table 2), the association between gluten intake and these standardized cognitive scores remained null. The null associations persisted when modeled gluten intake as a continuous variable (eTable 1 in the Supplement) or by decile categories (eTable 2 in the Supplement).

In stratified analyses by age, smoking status, BMI, depression status, and history of diabetes, hypertension, and hypercholesterolemia (Table 3), most of the interactions between gluten and these factors were null. However, the association between gluten intake and standardized psychomotor speed and attention score was potentially modified by smoking status, with higher gluten intake associated with slightly lower score among those who ever smoked (highest vs lowest quintile: −0.09; 95% CI, −0.18 to 0.01; *P* for trend = .02). However, this association became null after adjustment for refined grains (highest vs lowest quintile: −0.06; 95% CI, −0.17 to 0.04; *P* for trend = .09) (eTable 3 in the Supplement). Although the association between gluten intake and global cognition was potentially modified by smoking status and history of hypercholesterolemia, there was no difference in global cognition score across gluten quintiles in any stratum of smoking and hypercholesterolemia status (Table 3).

**Table 3.** Multivariable-Adjusted Differences in Standardized Cognitive Scores Associated With Quintiles of Gluten Intake, According to Different Strata^a^

Strata	No.	Difference by quintiles of gluten intake, mean (95% CI)							*P* value	
		1, lowest	2		3		4	5 (highest)	For trend^b^	For interaction^c^
**Psychomotor speed and attention**^d^										
Age, y										
<65	10 234	0 [Reference]	0.02 (−0.04 to 0.08)	0.03 (−0.03 to 0.09)		−0.03 (−0.09 to 0.03)		−0.02 (−0.08 to 0.04)	.15	.62
≥65	3260	0 [Reference]	−0.02 (−0.13 to 0.09)	−0.05 (−0.16 to 0.06)		−0.01 (−0.12 to 0.11)		0.004 (−0.11 to 0.12)	.90	
Smoking										
Never	8932	0 [Reference]	0.02 (−0.05 to 0.08)	0.02 (−0.05 to 0.08)		0.01 (−0.06 to 0.07)		0.01 (−0.05 to 0.08)	.88	.02
Ever	4550	0 [Reference]	0.003 (−0.09 to 0.09)	0.01 (−0.08 to 0.09)		−0.09 (−0.18 to 0.002)		−0.09 (−0.18 to 0.01)	.02	
BMI										
<25	7032	0 [Reference]	0.01 (−0.07 to 0.08)	0.002 (−0.07 to 0.08)		−0.06 (−0.14 to 0.01)		−0.05 (−0.12 to 0.02)	.05	.31
≥25	6462	0 [Reference]	0.01 (−0.06 to 0.09)	0.02 (−0.05 to 0.10)		0.02 (−0.06 to 0.09)		0.005 (−0.07 to 0.08)	.91	
Current depression status										
No	9217	0 [Reference]	0.001 (−0.06 to 0.06)	−0.004 (−0.07 to 0.06)		−0.01 (−0.07 to 0.05)		−0.02 (−0.09 to 0.04)	.43	.62
Yes	4277	0 [Reference]	0.03 (−0.07 to 0.13)	0.05 (−0.05 to 0.15)		−0.05 (−0.15 to 0.05)		−0.05 (−0.15 to 0.05)	.33	
History of diabetes										
No	12 503	0 [Reference]	0.02 (−0.04 to 0.07)	0.02 (−0.04 to 0.07)		−0.02 (−0.07 to 0.04)		−0.01 (−0.07 to 0.04)	.34	.41
Yes	991	0 [Reference]	−0.11 (−0.31 to 0.08)	−0.06 (−0.26 to 0.14)		−0.11 (−0.33 to 0.10)		−0.15 (−0.38 to 0.07)	.21	
History of hypertension										
No	8337	0 [Reference]	0.01 (−0.06 to 0.08)	0.01 (−0.06 to 0.08)		−0.05 (−0.12 to 0.02)		−0.04 (−0.11 to 0.03)	.07	.17
Yes	5157	0 [Reference]	0.02 (−0.07 to 0.10)	0.02 (−0.07 to 0.10)		0.02 (−0.07 to 0.11)		0.01 (−0.08 to 0.10)	.75	
History of hypercholesterolemia										
No	5616	0 [Reference]	−0.01 (−0.09 to 0.08)	−0.04 (−0.12 to 0.05)		−0.08 (−0.16 to −0.001)		−0.07 (−0.15 to 0.01)	.03	.10
Yes	7878	0 [Reference]	0.02 (−0.05 to 0.09)	0.04 (−0.02 to 0.11)		0.01 (−0.06 to 0.08)		0.01 (−0.07 to 0.08)	.99	
**Learning and working memory**^d^										
Age, y										
<65	10 234	0 [Reference]	0.02 (−0.03 to 0.08)	0.11 (0.05 to 0.16)		0.07 (0.01 to 0.13)		0.04 (−0.02 to 0.10)	.16	.19
≥65	3260	0 [Reference]	−0.01 (−0.12 to 0.09)	−0.01 (−0.11 to 0.10)		−0.04 (−0.14 to 0.07)		−0.004 (−0.11 to 0.10)	.80	
Smoking										
Never	8932	0 [Reference]	0.01 (−0.05 to 0.08)	0.06 (−0.01 to 0.12)		0.06 (−0.001 to 0.13)		0.03 (−0.04 to 0.09)	.24	.31
Ever	4550	0 [Reference]	0.02 (−0.07 to 0.10)	0.10 (0.02 to 0.19)		−0.01 (−0.09 to 0.08)		0.01 (−0.08 to 0.10)	.99	
BMI										
<25	7032	0 [Reference]	−0.02 (−0.09 to 0.06)	0.06 (−0.02 to 0.13)		0.05 (−0.02 to 0.12)		−0.001 (−0.07 to 0.07)	.62	.78
≥25	6462	0 [Reference]	0.04 (−0.03 to 0.12)	0.10 (0.02 to 0.17)		0.02 (−0.05 to 0.10)		0.05 (−0.03 to 0.13)	.33	
Current depression status										
No	9217	0 [Reference]	−0.01 (−0.07 to 0.05)	0.06 (0.003 to 0.12)		0.06 (0.0002 to 0.12)		0.03 (−0.03 to 0.09)	.10	.13
Yes	4277	0 [Reference]	0.06 (−0.04 to 0.15)	0.09 (−0.001 to 0.19)		0.005 (−0.09 to 0.10)		0.01 (−0.09 to 0.10)	.65	
History of diabetes										
No	12 503	0 [Reference]	0.02 (−0.03 to 0.07)	0.08 (0.03 to 0.14)		0.05 (−0.003 to 0.10)		0.03 (−0.03 to 0.08)	.26	.63
Yes	991	0 [Reference]	−0.07 (−0.26 to 0.12)	−0.03 (−0.22 to 0.16)		−0.06 (−0.27 to 0.15)		−0.01 (−0.22 to 0.21)	.95	
History of hypertension										
No	8337	0 [Reference]	0.001 (−0.07 to 0.07)	0.07 (0.002 to 0.13)		0.04 (−0.03 to 0.10)		0.02 (−0.04 to 0.09)	.36	.94
Yes	5157	0 [Reference]	0.03 (−0.05 to 0.11)	0.08 (0.001 to 0.16)		0.05 (−0.04 to 0.13)		0.02 (−0.07 to 0.11)	.57	
History of hypercholesterolemia										
No	5616	0 [Reference]	−0.08 (−0.16 to 0.005)	−0.03 (−0.11 to 0.05)		−0.03 (−0.11 to 0.05)		−0.06 (−0.14 to 0.02)	.45	.07
Yes	7878	0 [Reference]	0.07 (0.01 to 0.14)	0.14 (0.08 to 0.21)		0.08 (0.02 to 0.15)		0.07 (0.004 to 0.14)	.04	
**Global cognition**^d^										
Age, y										
<65	10 234	0 [Reference]	0.03 (−0.03 to 0.09)	0.08 (0.02 to 0.14)		0.02 (−0.04 to 0.08)		0.004 (−.06 to 0.06)	.84	.72
≥65	3260	0 [Reference]	−0.02 (−0.13 to 0.08)	−0.04 (−0.14 to 0.07)		−0.03 (−0.14 to 0.08)		0.0001 (−0.11 to 0.11)	.96	
Smoking										
Never	8932	0 [Reference]	0.02 (−0.05 to 0.08)	0.04 (−0.02 to 0.11)		0.04 (−0.03 to 0.10)		0.02 (−0.04 to 0.09)	.46	.03
Ever	4550	0 [Reference]	0.01 (−0.07 to 0.10)	0.06 (−0.03 to 0.15)		−0.06 (−0.15 to 0.03)		−0.05 (−0.15 to 0.04)	.10	
BMI										
<25	7032	0 [Reference]	−0.003 (−0.08 to 0.07)	0.03 (−0.04 to 0.10)		−0.01 (−0.09 to 0.06)		−0.03 (−0.10 to 0.04)	.29	.41
≥25	6462	0 [Reference]	0.03 (−0.04 to 0.11)	0.07 (−0.01 to 0.14)		0.02 (−0.05 to 0.10)		0.03 (−0.05 to 0.11)	.55	
Current depression status										
No	9217	0 [Reference]	−0.005 (−0.06 to 0.06)	0.03 (−0.03 to 0.09)		0.03 (−0.04 to 0.09)		0.002 (−0.06 to 0.06)	.73	.25
Yes	4277	0 [Reference]	0.05 (−0.05 to 0.15)	0.08 (−0.01 to 0.18)		−0.03 (−0.13 to 0.07)		−0.01 (−0.11 to 0.09)	.37	
History of diabetes										
No	12 503	0 [Reference]	0.02 (−0.03 to 0.08)	0.06 (0.004 to 0.11)		0.01 (−0.04 to 0.07)		0.01 (−0.05 to 0.06)	.96	.41
Yes	991	0 [Reference]	−0.11 (−0.31 to 0.08)	−0.06 (−0.25 to 0.13)		−0.11 (−0.32 to 0.10)		−0.11 (−0.32 to 0.11)	.37	
History of hypertension										
No	8337	0 [Reference]	0.01 (−0.06 to 0.07)	0.04 (−0.02 to 0.11)		−0.01 (−0.08 to 0.05)		−0.01 (−0.08 to 0.05)	.47	.34
Yes	5157	0 [Reference]	0.03 (−0.05 to 0.11)	0.06 (−0.03 to 0.14)		0.04 (−0.05 to 0.13)		0.02 (−0.07 to 0.11)	.61	
History of hypercholesterolemia										
No	5616	0 [Reference]	−0.05 (−0.13 to 0.04)	−0.04 (−0.12 to 0.04)		−0.07 (−0.15 to 0.01)		−0.07 (−0.15 to 0.01)	.06	.04
Yes	7878	0 [Reference]	0.05 (−0.01 to 0.12)	0.11 (0.04 to 0.17)		0.05 (−0.02 to 0.12)		0.04 (−0.03 to 0.11)	.28	

Abbreviation: BMI, body mass index (calculated as weight in kilograms divided by height in meters squared).

^a^ Energy-adjusted gluten intake was cumulatively averaged from 1991 to the last questionnaire cycle preceding the cognitive assessment. Model was adjusted for age, race, body mass index, husband’s educational attainment, family income, history of diabetes, history of hypertension, history of hypercholesterolemia, history of myocardial infarction, current depression status, smoking, aspirin or nonsteroid anti-inflammatory drug use, multivitamins use, physical activity, menopausal status and hormone use, total energy intake, alcohol intake, and Alternative Healthy Eating Index score, excluding alcohol and whole grains.

^b^ *P* value for trend was calculated using the median value for each gluten intake quintile as a continuous variable.

^c^ *P* value for interaction was estimated using an interaction term of gluten intake and the respective stratifying variable.

^d^ Standardized scores for psychomotor speed and attention, learning and working memory, and global cognition were calculated by standardizing the mean standardized scores of the following Cogstate battery tasks: Detection and Identification, One Card Learning and One Back, and all 4 four tasks, respectively. Higher scores indicate better performance, with 1 unit increase representing 1 SD higher than the mean.

In a sensitivity analysis of women who had never reported cancer diagnosis (eTable 4 in the Supplement), never reported dementia diagnosis (eTable 5 in the Supplement), or had completed all previous dietary assessments (eTable 6 in the Supplement), the null associations between gluten intake and each cognitive scores were similar to the main model. When examining gluten intake estimated during each 4-year questionnaire cycle prior to cognitive assessment (eTable 7 in the Supplement), distant past (12-24 years), and recent past (4-12 years), and change in gluten intake from distant to recent past (eTable 8 in the Supplement), the null associations between gluten intake and cognitive scores remained.

## Discussion

In this cohort of middle-aged women without celiac disease, we found no evidence of a meaningful association between long-term gluten intake during 2 decades and subsequent cognitive performance on a validated cognitive battery. Short-term gluten intake and change in gluten intake were likewise not associated with cognitive performance.

The prevalence of gluten avoidance in the general population without celiac disease has markedly increased in the past decade.^7,42^ Many factors contribute to the popularity of a gluten-free diet, including its touted effects on weight loss, metabolic syndrome, and intestinal symptoms.^10,43,44^ However, many of these putative benefits lack consistent evidence.^7,45^ In fact, several studies have raised nutritional concerns, given that some gluten-free foods tend to contain less protein and fiber but higher saturated lipids and sodium than non–gluten-free foods.^46,47^ To account for potential differences in diet quality associated with different levels of gluten intake, we adjusted for the AHEI-10 score in our model. The null associations between gluten and cognitive scores remained after subsequent adjustment for refined gains or whole grains, which are major sources of dietary gluten, indicating that these null associations were independent of diet quality and gluten sources.

Recently, the concept that gluten could affect cognitive function was popularized by a best-selling book, *Grain Brain*.^10^ Concern for such an association is derived in part from patients with celiac disease. Subjective cognitive symptoms, referred to colloquially as brain fog, are often reported by patients with celiac disease preceding diagnosis and after suspected accidental gluten exposure,^5^ although this symptom has not been formally defined as a medical condition because of limited empirical evidence. Additionally, a large, prospective, population-based study found that celiac disease was not associated with risk for Alzheimer dementia over a median follow-up of 8.4 years, but it was associated with a slight increase in risk for vascular dementia.^48^

However, current findings on the association between dietary gluten and cognition in celiac disease are inconsistent. In a study of 11 patients aged 22 to 39 years who had been recently diagnosed with celiac disease, Lichtwark et al^6^ found that, in parallel to mucosal healing, cognitive performance was improved after following a gluten-free diet for 52 weeks. In contrast, a case-control study found that despite more than 1 to 12 years of gluten avoidance, 18 patients with celiac disease older than 65 years had worse cognitive performance compared with 18 age- and-sex-matched controls.^49^ These results suggest that the possible ameliorating effects of gluten avoidance on cognition may be related to intestinal health and modified by age. In our stratified analyses, we found that the null associations between gluten intake and cognitive scores were not different by age above or below 65 years. Instead, we observed a potential effect modification by smoking status, with higher gluten intake associated with lower psychomotor speed/attention score among those who ever smoked. However, this association became null after adjustment for refined gain consumption. Given that the differences in cognitive scores across the quintiles of gluten intake were extremely small (ie, <0.1 SD) and a total of 21 interactions were tested, these results are unlikely to be clinically meaningful, subject to multiple testing, and should be verified in future study.

Despite the lack of evidence on the gluten-cognition association among individuals without celiac disease or nonceliac gluten sensitivity, proponents of a wider effect of gluten beyond celiac disease have hypothesized that wheat may play a role as an exorphin, exerting central opioidlike effects and thus affecting memory and mood.^50,51^ Our results, based on a large population-based cohort, does not support this theory and suggest that gluten intake is not associated with cognitive decline, which is in line with the recent position of the Global Council on Brain Health.^52^

There are several strengths in our study, including its large sample size, high follow-up rate, long-term dietary gluten intake measured prospectively and independently of the primary outcome, and the use of a validated cognitive battery.^25,26^ In addition, numerous sensitivity analyses that tested gluten intake at different time periods and change in gluten intake confirmed the robustness of our findings by consistently showing a null association between gluten and cognition.

### Limitations

This study has limitations. First, the study population consisted of women at midlife from a relatively homogeneous educational and socioeconomic background, potentially limiting its generalizability to other populations. In addition, we had a single measure of cognitive function, which may not adequately represent cognitive trajectories over time, and thus, future research with repeated measures is merited. Furthermore, our FFQs did not ask specifically about gluten-free products or adherence to a gluten-free diet and thus may overestimate gluten intake among participants who selectively consumed such products. Moreover, gluten was calculated based on the estimated percentages of protein in commonly consumed foods containing wheat, rye, and barley. Although this method is widely accepted and has been validated using 1-week diet records,^12,13,14,16,22,23^ it is insufficiently sensitive to identify individuals consuming a gluten-free diet, and thus, our conclusions should not be extrapolated to apply to that population. Nevertheless, our results showed no significant difference in cognitive scores when comparing the highest decile (median, 9.04 g/d) with the lowest decile (median, 3.97 g/d) of gluten intake. Furthermore, as an observational study, we cannot rule out the possibility of unmeasured residual confounding. However, we adjusted for many potential confounding factors, and multivariate adjustment did not materially alter our age-adjusted estimates.

## Conclusions

In this study, we found no significant association of long-term, short-term, or change in gluten intake with cognitive function among women without celiac disease. Our findings suggest that restricting dietary gluten for the purpose of maintaining or improving cognition is not warranted in the absence of celiac disease or established gluten sensitivity. Further longitudinal research in more diverse population is needed to confirm our findings.

## Notes

Supplement. **eMethods.** Supplementary Methods **eTable 1.** Mean Differences in Standardized Cognitive Scores Associated With Continuous Gluten Intake in Grams per Day Among 13 494 Women **eTable 2.** Mean Differences in Standardized Cognitive Scores Associated With Deciles of Gluten Intake Among 13 494 Women **eTable 3.** Multivariable-Adjusted Differences in Standardized Psychomotor Speed and Attention Score Across Quintiles of Gluten Intake by Smoking Status With Additional Adjustment for Refined Grains Among 13 494 Women **eTable 4.** Sensitivity Analysis of the Mean Differences in Standardized Cognitive Scores Associated With Quintiles of Gluten Intake Among 11 733 Women Who Had Never Reported Cancer Diagnosis **eTable 5.** Sensitivity Analysis of the Mean Differences in Standardized Cognitive Scores Associated With Quintiles of Gluten Intake Among 13 486 Women Who Had Never Reported Dementia Diagnosis **eTable 6.** Sensitivity Analysis of the Mean Differences in Standardized Cognitive Scores Associated With Quintiles of Gluten Intake Among 11 646 Women Who Completed All Food Frequency Questionnaires During 24-Year Follow-up Prior to Cognitive Assessment **eTable 7. **Multivariable-Adjusted Mean Differences in Standardized Cognitive Scores Associated With Quintiles of Gluten Intake by 4-Year Interval Prior to Cognitive Assessment Among 11 646 Women **eTable 8.** Multivariable-Adjusted Mean Differences in Standardized Cognitive Scores Associated With 1 SD (1.98 g/d) Increase in Mean Gluten Intake From Distant Past (12-24 y) to Recent Past (4-12 y) of Cognitive Assessment Among 11 646 Women **eFigure 1.** Nurses’ Health Study II Questionnaire in 2015 Questionnaire Cycle **eFigure 2.** Study Flow Diagram eReferences.
