# Supplementary material for: Long-term Intake of Gluten and Cognitive Function Among US Women
Source: JAMA Netw Open. 2021 May 21;4(5):e2113020. doi: 10.1001/jamanetworkopen.2021.13020 (PMC8140370; doi:10.1001/jamanetworkopen.2021.13020)
Supplement: Supplement. — eMethods. Supplementary Methods eTable 1. Mean Differences in Standardized Cognitive Scores Associated With Continuous Gluten Intake in Grams per Day Among 13 494 Women eTable 2. Mean Differences in Standardized Cognitive Scores Associated With Deciles of Gluten Intake Among 13 494 Women eTable 3. Multivariable-Adjusted Differences in Standardized Psychomotor Speed and Attention Score Across Quintiles of Gluten Intake by Smoking Status With Additional Adjustment for Refined Grains Among 13 494 Women eTable 4. Sensitivity Analysis of the Mean Differences in Standardized Cognitive Scores Associated With Quintiles of Gluten Intake Among 11 733 Women Who Had Never Reported Cancer Diagnosis eTable 5. Sensitivity Analysis of the Mean Differences in Standardized Cognitive Scores Associated With Quintiles of Gluten Intake Among 13 486 Women Who Had Never Reported Dementia Diagnosis eTable 6. Sensitivity Analysis of the Mean Differences in Standardized Cognitive Scores Associated With Quintiles of Gluten Intake Among 11 646 Women Who Completed All Food Frequency Questionnaires During 24-Year Follow-up Prior to Cognitive Assessment eTable 7. Multivariable-Adjusted Mean Differences in Standardized Cognitive Scores Associated With Quintiles of Gluten Intake by 4-Year Interval Prior to Cognitive Assessment Among 11 646 Women eTable 8. Multivariable-Adjusted Mean Differences in Standardized Cognitive Scores Associated With 1 SD (1.98 g/d) Increase in Mean Gluten Intake From Distant Past (12-24 y) to Recent Past (4-12 y) of Cognitive Assessment Among 11 646 Women eFigure 1. Nurses’ Health Study II Questionnaire in 2015 Questionnaire Cycle eFigure 2. Study Flow Diagram eReferences. [file jamanetwopen-e2113020-s001.pdf]

Supplemental Online Content

Wang Y, Lebwohl B, Mehta R, et al. Long-term intake of gluten and cognitive function among US women. *JAMA Netw Open*. 2021;4(5):e2113020. doi:10.1001/jamanetworkopen.2021.13020

eMethods. Supplementary Methods

eTable 1. Mean Differences in Standardized Cognitive Scores Associated With Continuous Gluten Intake in Grams per Day Among 13 494 Women

eTable 2. Mean Differences in Standardized Cognitive Scores Associated With Deciles of Gluten Intake Among 13 494 Women

eTable 3. Multivariable-Adjusted Differences in Standardized Psychomotor Speed and Attention Score Across Quintiles of Gluten Intake by Smoking Status With Additional Adjustment for Refined Grains Among 13 494 Women

eTable 4. Sensitivity Analysis of the Mean Differences in Standardized Cognitive Scores Associated With Quintiles of Gluten Intake Among 11 733 Women Who Had Never Reported Cancer Diagnosis

eTable 5. Sensitivity Analysis of the Mean Differences in Standardized Cognitive Scores Associated With Quintiles of Gluten Intake Among 13 486 Women Who Had Never Reported Dementia Diagnosis

eTable 6. Sensitivity Analysis of the Mean Differences in Standardized Cognitive Scores Associated With Quintiles of Gluten Intake Among 11 646 Women Who Completed All Food Frequency Questionnaires During 24-Year Follow-up Prior to Cognitive Assessment

eTable 7. Multivariable-Adjusted Mean Differences in Standardized Cognitive Scores Associated With Quintiles of Gluten Intake by 4-Year Interval Prior to Cognitive Assessment Among 11 646 Women

eTable 8. Multivariable-Adjusted Mean Differences in Standardized Cognitive Scores Associated With 1 SD (1.98 g/d) Increase in Mean Gluten Intake From Distant Past (12-24 y) to Recent Past (4-12 y) of Cognitive Assessment Among 11 646 Women

eFigure 1. Nurses' Health Study II Questionnaire in 2015 Questionnaire Cycle

eFigure 2. Study Flow Diagram

eReferences.

This supplemental material has been provided by the authors to give readers additional information about their work.

© 2021 Wang Y et al. *JAMA Network Open.*

eMethods. Supplementary Methods

Dietary assessment

Diet was assessed in 1991, 1995, 1999, 2003, 2007, 2011 and 2015 in Nurses' Health Study II (NHSII) using a semi-quantitative food frequency questionnaire (FFQ, eFigure 1). Participants were asked how frequently they consumed each food item in common portion size during the past year, with nine possible responses ranged from "Never or less than once per month" to "6+ per day".

To estimate servings of food groups consumed per day, we first converted the FFQ responses to servings/day and then summed up all food items in each food group. For example, whole grains included oats, brown rice, dark bread, whole-grain cracker, whole-grain cereal, bran, and wheat germ, while refined grains included white bread, white rice, muffin, pancake, pasta, and tortilla. We used the Harvard T. H. Chan School of Public Health nutrient database updated during each questionnaire cycle to calculate nutrient intakes. Previous validation studies in men and women have demonstrated comparable reproducibility (intraclass correlation coefficients: 0.49-0.71 in women) and modest correlation (Spearman correlation coefficient r: 0.36-0.75 in women) between FFQs and one-week food diaries.[1, 2]

Specifically, for gluten intake, we first identified food items with gluten-containing ingredients like wheat, wheat bran, wheat germ, rye, barley, cereal, and pasta, according to the ingredient lists on commercial product labels provided by manufacturers and recipes on cookbooks for home-cooked items. Then, we quantified the gluten content of the gluten-containing ingredients in all food item by multiplying the protein content of these ingredients with an approximate proportion of gluten (75%), in line with previous studies,[3, 4] though the proportion of gluten in protein may be more variable in barley and rye than wheat.[5] We did not calculate the trace amount of gluten in oats and certain condiments like soy sauce since the quantity of gluten in these foods would be negligible compared to the total gluten intake.[6] Next, we added up gluten from all foods consumed to estimate total gluten intake.

In a recent validation study using 7-day dietary records, FFQ-derived gluten intake showed moderate to high validity (r: 0.58 for gluten, median r: 0.60 for gluten-rich foods).[7] Another validation studies also showed reasonable correlation between FFQ-derived and 7-day diet records-derived major sources of gluten (e.g., r: 0.79 for cold cereal).[8] Zong et al. has reported the trend of gluten intake over the past two decades in NHSII and major sources of gluten consumed in 2011, including whole-grain bread, pasta, cold cereal, pizza, and wheat products like pretzel, bagel, muffin, crackers, white bread, and tortilla.[4]

To account for confounding from total energy intake and potential under- or over-reporting, we adjusted gluten intake for total energy intake using the residual method. As previously described,[9] absolute nutrient intake was regressed on total energy intake to get the nutrient residual, which was uncorrelated with the total energy intake, allowing direct evaluation of the variation in nutrient composition. To estimate long-term intakes, we averaged across FFQs preceding the cognitive assessment. For participants who completed cognitive assessment before the 2015 questionnaire cycle, we averaged daily gluten intake reported in 1991, 1995, 1999, 2003, 2007, and 2011. For the rest of participants, we averaged daily gluten intake reported in 1991, 1995, 1999, 2003, 2007, 2011, and 2015. We categorized participants into quintiles of energy adjusted gluten intake (0.1-<5.1, 5.1-<5.9, 5.9-<6.6, 6.6-<7.6,7.6-<18.3 g/day) to assess potential non-linear relationships and threshold of effect.

Cognitive assessment

We used the CogState Brief Battery to assess cognitive function of participants.[10] The CogState Brief Battery was self-administered at home via a web-based interface and took about 15-20 minutes to complete.[10] As the battery were loaded from Internet and ran locally on participants' computers, the cognitive performance would not be affected by Internet connection and speed. Hardware differences would cause minimal time variation as well (on the order of milliseconds).[11] The validity of this unsupervised online battery has been well-demonstrated,[12-14] and its utility has been evaluated in large population-based epidemiological studies.[10, 11] Moreover, the CogsState battery was sensitive in detecting mild cognitive impairment[12, 15] and had comparable performance in supervised and unsupervised settings.[14]

© 2021 Wang Y et al. *JAMA Network Open.*

The CogState Brief Battery contained four tasks in the following order, all involved images of playing cards: Detection, Identification, One Card Learning, and One Back.[10] Participants had to view instructions and complete a practice trial before each task started. During each task, participants responded to playing cards by pressing the "K" and "D" computer keys, which meant "Yes" and "No", respectively. As previously described,[10-14] in Detection, which assessed psychomotor function and information processing speed, participants pressed the "K" key as quickly as possible when a joker card turned face-up. In Identification, which assessed visual attention and vigilance, participants pressed the "K" or "D" key as quickly as possible to indicate whether the faced-up joker card was red or not. In One Card Learning, which assessed visual recognition memory and attention, participants responded "K" or "D" if the faced-up card (normal playing card without joker) had appeared in the task before or not. In One Back, which assessed working memory and attention, participants responded "K" or "D" if the faced-up normal playing card was the same as the immediately previous card or not. The speed (mean reaction time in milliseconds) and accuracy (proportion correct) were recorded for each task. The performance on Detection, Identification, and One Back was measured based on the $\log_{10}$ transformed reaction time for correct responses. These scores were then standardized by z-normalization and reversed so that a higher score indicated better performance. The performance on One Card Learning was measured based on arcsine square-root transformed proportion of correct responses. This score was also standardized using z-normalization. A validation study by Lim et al. found that patients with mild cognitive impairment and Alzheimer's disease had greatest impairment in memory (One Card Learning and One Back) and apolipoprotein E4 genotype had negative influence on performance in One Card Learning.[15]

Consistent with previous studies,[11-14] we computed composite scores on these tasks as these scores may be more sensitive to detect cognitive variations than individual task scores. We averaged the z-scores on Detection and Identification to calculate psychomotor speed/attention score, averaged the z-scores on One Card Learning and One Back to calculate learning/working memory score, and averaged the z-scores on all four tasks to calculate the global cognition score. These composite scores were then re-standardized using z-normalization so that one unit increase in score indicated 1 SD higher than the sample mean. The composite score was not computed if any of the contributing task scores was missing. We excluded implausibly low score based on cutoffs of percent correctness for each task recommended by CogState (Detection: <0.8; Identification: <0.8; One Card Learning: <0.5; One Back: <0.7), because they were likely due to technical errors. These composite scores have been shown to have high test-retest reliability (r=0.95) in identifying adults with cognitive impairment.[12] Sumner et al. has shown that the standardized loadings for responses times on Detection (0.68) and Identification (0.95) for the psychomotor speed/attention factor and the standardized loadings for responses times on One Card Learning (0.69) and One Back (0.85) for the learning/working memory factor were high in a confirmatory factor analysis of NHSII cohort,[11] indicating that these composite scores were applicable in our cohort.

Covariates

Participants self-reported date of birth, race (White/non-White), husband's education (high school or below, college, graduate, unmarried or missing), family income (dollar), personal history of diseases (hypertension, diabetes, hypercholesterolemia, myocardial infarction, depression, cancer, and dementia), body mass index (BMI, kg/m^2), smoking status (never, past, current, and missing n=12), regular use of medications (antidepressant, aspirin, non-steroidal anti-inflammatory drugs, postmenopausal hormone), use of multivitamin, and alcohol intake (g/day) in questionnaires. We averaged BMI, physical activity, total energy intake, and alcohol intake across questionnaire cycles from 1991 to the most recent questionnaire (2011, 2013, or 2015) prior to cognitive assessment, and acquired other covariates from the most recent questionnaire prior to cognitive assessment. Husband's education was measured in 1999 (missing n=391). We used husband's education as an indicator for socioeconomic status because participants in NHSII were nurses with similar education background. We included family income as a covariate to additional capture another domain of socioeconomic status of participants, since it has been found to be associated with diet quality and cognitive function.[16, 17]

We assessed current depression status based on self-reported depression diagnosis, treatment, and clinically relevant depressive symptoms (≥10) according to the validated 10-item Centre for Epidemiological Studies Depression Scale (CES-D-10), which was assessed in 2013. The CES-D-10 includes 3, 5, and 2 questions on depressed effect, somatic symptoms, and positive affect in the past week, respectively, with options ranging from "rarely or none of the time", which contributes 0 score, to "all of the time", which contributes 3 scores.[18] We included depression status as a covariate because it has been shown that depression was associated with cognitive deficits.[19]

© 2021 Wang Y et al. *JAMA Network Open.*

We computed the Alternative Health Eating Index 2010 (AHEI-10) as described previously,[20] without alcohol and whole grain components. The included AHEI-10 components each assigned a score of 0-10, with higher scores for higher intakes of vegetables, fruits, nuts and legumes, long-chain (n-3) fatty acids, and poly-unsaturated fatty acids, but lower scores for higher intakes of fruit juice and sugar-sweetened beverages, red and processed meats, trans fats, and sodium.[20] This score has been widely used in NHSII studies to assess diet quality[4, 11] and was strongly associated with major chronic diseases like coronary heart disease, diabetes, and cancer.[20] Like other dietary variables, we averaged AHEI-10 scores across previous questionnaires.

For 22, 5, and 8 participants with missing information on family income, BMI, and AHEI-10, respectively, we imputed missing values with median values of these variables. For 2 participants with missing information on aspirin or non-steroidal anti-inflammatory drug use, we carried forward the last non-missing response.

Statistical analysis

We included the above covariates in our multivariable-adjusted linear regression model examining mean difference in each standardized cognitive score comparing across quintiles of gluten intake. We used a linear trend analysis to test the overall significance of the gluten variable and whether the cognitive score increased or decreased across gluten quintiles, by assigning the median quintile value to each gluten category (4.45 g/day, 5.48 g/day, 6.23 g/day, 7.03 g/day, 8.34 g/day) and testing this variable as a continuous variable. This trend test has been widely used in prior analyses of data from our cohort.[3, 4, 20, 21] To test the robustness of this trend test, we modeled the continuous gluten intake variable in g/day to test its linear relationships with cognitive scores.

To assess whether gluten intake during different time periods relative to the cognitive assessment would produce different results, we first calculated gluten intake assessed during each 4-year FFQ cycle (≤4 years, 4-8 years, 8-12 years, 12-16 years, 16-20 years, and 20-24 years before cognitive assessment), average gluten intake in distant past (12-24 years), and average gluten intake in recent past (4-12 years), and then used each of these gluten variables in the linear model as the main exposure. To test whether change in gluten intake over time would affect cognitive function, we calculated the change in gluten intake from distant to recent past (mean: -0.87 g/day) and examined the mean difference in each cognitive score associated with per 1 SD (1.98 g/day) increase in gluten intake.

Other statistical analyses have been described in detail in the Methods section of main texts.

© 2021 Wang Y et al. *JAMA Network Open.*

eTable 1. Mean Differences in Standardized Cognitive Scores Associated With Continuous Gluten Intake in Grams per Day Among 13 494 Women

Composite cognitive scores[a]	Age-adjusted		Multivariable-adjusted[b]	
	Estimate (95% confidence interval)	*P*	Estimate (95% confidence interval)	*P*
Psychomotor speed/attention	-0.003 (-0.01, 0.01)	0.63	-0.01 (-0.02, 0.004)	0.23
Learning/working memory	0.01 (-0.00001, 0.02)	0.05	0.005 (-0.01, 0.02)	0.39
Global cognition	0.004 (-0.01, 0.01)	0.47	-0.002 (-0.01, 0.01)	0.73

[a] Standardized scores for psychomotor speed/attention, learning/working memory, and global cognition were calculated by standardizing as the mean of the standardized scores of the following CogState battery tasks: Detection + Identification, One Card Learning + One-Back, and all four tasks, respectively. Higher scores indicate better performance, with one unit increase represents 1 SD higher than the mean.

[b] Energy-adjusted gluten intake was cumulatively averaged from 1991 to the last questionnaire cycle preceding the cognitive assessment. Model was adjusted for age (years), race (white, non-white), body mass index (kg/m^2), husband's educational attainment (high school or below, college, graduate school, unmarried/missing), family income (dollar), history of diabetes (yes, no), history of hypertension (yes, no), history of hypercholesterolemia (yes, no), history of myocardial infarction (yes, no), current depression status (yes, no), smoking (never, past, current, missing), aspirin or non-steroid anti-inflammatory drug use (yes, no), multivitamins use (yes, no), physical activity (metabolic equivalents/week), menopausal status and hormone use (pre-menopause, post-menopause and never/past/current user of hormone therapy), total energy intake (kcal/d), alcohol intake (g/d), and Alternative Healthy Eating Index score (excluding alcohol and whole grains).

© 2021 Wang Y et al. *JAMA Network Open.*

**eTable 2. Mean Differences in Standardized Cognitive Scores Associated With Deciles of Gluten Intake Among 13 494 Women[a]**											
**Model**	**Deciles of average gluten intake**										***P*-trend[b]**
	**Q1 (lowest)**	**Q2**	**Q3**	**Q4**	**Q5**	**Q6**	**Q7**	**Q8**	**Q9**	**Q10 (highest)**	
**Median gluten intake (g/day)**	3.97	4.79	5.28	5.68	6.04	6.41	6.82	7.29	7.90	9.04	--
**Psychomotor speed/attention[c]**											
Age-adjusted	Ref.	0.02 (-0.06, 0.09)	0.005 (-0.07, 0.08)	0.04 (-0.03, 0.12)	0.03 (-0.05, 0.10)	0.03 (-0.04, 0.10)	0.01 (-0.07, 0.08)	-0.02 (-0.09, 0.05)	0.02 (-0.06, 0.09)	-0.01 (-0.08, 0.06)	0.52
Multivariable-adjusted[d]	Ref.	0.01 (-0.06, 0.09)	-0.002 (-0.08, 0.07)	0.03 (-0.04, 0.11)	0.02 (-0.06, 0.09)	0.02 (-0.06, 0.09)	-0.0002 (-0.07, 0.07)	-0.04 (-0.11, 0.04)	0.005 (-0.07, 0.08)	-0.03 (-0.11, 0.04)	0.18
**Learning/working memory[c]**											
Age-adjusted	Ref.	0.01 (-0.06, 0.09)	0.01 (-0.06, 0.08)	0.05 (-0.02, 0.13)	0.09 (0.01, 0.16)	0.11 (0.04, 0.18)	0.09 (0.01, 0.16)	0.05 (-0.02, 0.12)	0.06 (-0.01, 0.14)	0.05 (-0.03, 0.12)	0.05
Multivariable-adjusted[d]	Ref.	-0.00 (-0.07, 0.07)	-0.01 (-0.08, 0.06)	0.03 (-0.04, 0.11)	0.06 (-0.01, 0.14)	0.08 (0.01, 0.15)	0.06 (-0.01, 0.13)	0.02 (-0.05, 0.09)	0.03 (-0.04, 0.11)	0.01 (-0.06, 0.08)	0.38
**Global cognition[c]**											
Age-adjusted	Ref.	0.02 (-0.05, 0.09)	0.01 (-0.06, 0.08)	0.06 (-0.02, 0.13)	0.06 (-0.01, 0.14)	0.08 (0.01, 0.15)	0.05 (-0.02, 0.13)	0.01 (-0.06, 0.09)	0.05 (-0.03, 0.12)	0.02 (-0.06, 0.09)	0.54
Multivariable-adjusted[d]	Ref.	0.01 (-0.07, 0.08)	-0.01 (-0.08, 0.07)	0.04 (-0.03, 0.11)	0.05 (-0.03, 0.12)	0.06 (-0.02, 0.13)	0.03 (-0.04, 0.11)	-0.01 (-0.09, 0.06)	0.02 (-0.05, 0.09)	-0.02 (-0.09, 0.06)	0.66

[a] Energy-adjusted gluten intake was cumulatively averaged from 1991 to the last questionnaire cycle preceding the cognitive assessment.

[b] P-trend was calculated using the median gluten intake for each decile as a continuous variable.

[c] Standardized scores for psychomotor speed/attention, learning/working memory, and global cognition were calculated by standardizing as the mean of the standardized scores of the following CogState battery tasks: Detection + Identification, One Card Learning + One-Back, and all four tasks, respectively. Higher scores indicate better performance, with one unit increase represents 1 SD higher than the mean.

[d] Model was adjusted for age (years), race (white, non-white), body mass index (kg/m2), husband's educational attainment (high school or below, college, graduate school, unmarried/missing), family income (dollar), history of diabetes (yes, no), history of hypertension (yes, no), history of hypercholesterolemia (yes, no), history of myocardial infarction (yes, no), current depression status (yes, no), smoking (never, past, current, missing), aspirin or non-steroid anti-inflammatory drug use (yes, no), multivitamins use (yes, no), physical activity (metabolic equivalents/week), menopausal status and hormone use (pre-menopause, post-menopause and never/past/current user of hormone therapy), total energy intake (kcal/d), alcohol intake (g/d), and Alternative Healthy Eating Index score (excluding alcohol and whole grains).

© 2021 Wang Y et al. *JAMA Network Open.*

eTable 3. Multivariable-Adjusted Differences in Standardized Psychomotor Speed and Attention Score Across Quintiles of Gluten Intake by Smoking Status With Additional Adjustment for Refined Grains Among 13 494 Women[a]

Strata	n	Quintiles of gluten intake					*P*-trend[b]	*P*-interaction[c]
		Q1 (lowest)	Q2	Q3	Q4	Q5 (highest)		
Women who never smoked	8,932	Ref.	0.02 (-0.05, 0.08)	0.02 (-0.04, 0.09)	0.01 (-0.05, 0.08)	0.02 (-0.05, 0.09)	0.69	0.02
Women who ever smoked	4,550	Ref.	0.01 (-0.08, 0.10)	0.02 (-0.07, 0.11)	-0.07 (-0.17, 0.02)	-0.06 (-0.17, 0.04)	0.09	

[a] Energy-adjusted gluten intake was cumulatively averaged from 1991 to the last questionnaire cycle preceding the cognitive assessment. Model was adjusted for age (years), race (white, non-white), body mass index (kg/m^2), husband's educational attainment (high school or below, college, graduate school, unmarried/missing), family income (dollar), history of diabetes (yes, no), history of hypertension (yes, no), history of hypercholesterolemia (yes, no), history of myocardial infarction (yes, no), current depression status (yes, no), smoking (never, past, current, missing), aspirin or non-steroid anti-inflammatory drug use (yes, no), multivitamins use (yes, no), physical activity (metabolic equivalents/week), menopausal status and hormone use (pre-menopause, post-menopause and never/past/current user of hormone therapy), total energy intake (kcal/d), alcohol intake (g/d), Alternative Healthy Eating Index score (excluding alcohol and whole grains), and refined grain intake. Standardized score for psychomotor speed and attention was calculated by standardizing the mean standardized scores of two CogState battery tasks, Detection and Identification. A higher score indicated better performance, with one unit increase representing 1 SD higher than the mean.

[b] *P*-trend was calculated using the median gluten intake for each quintile as a continuous variable.

[c] *P*-interaction was estimated using an interaction term of gluten intake and the respective stratifying variable.

© 2021 Wang Y et al. *JAMA Network Open.*

eTable 4. Sensitivity Analysis of the Mean Differences in Standardized Cognitive Scores Associated With Quintiles of Gluten Intake Among 11 733 Women Who Had Never Reported Cancer Diagnosis[a]

Model	Quintiles of average gluten intake					*P*-trend[b]
	Q1 (lowest)	Q2	Q3	Q4	Q5 (Highest)	
**Psychomotor speed/attentionv[c]**						
Standardized score mean (SD)	-0.02 (1.03)	0.01 (1.00)	0.03 (0.96)	0.01 (0.99)	0.03 (0.99)	--
Age-adjusted	Ref.	0.02 (-0.04, 0.07)	0.02 (-0.04, 0.08)	-0.004 (-0.06, 0.05)	0.005 (-0.05, 0.06)	0.86
Multivariable-adjusted[d]	Ref.	0.01 (-0.04, 0.07)	0.01 (-0.04, 0.07)	-0.02 (-0.07, 0.04)	-0.01 (-0.07, 0.05)	0.44
**Learning/working memory[c]**						
Standardized score mean (SD)	-0.05 (0.98)	0.01 (0.96)	0.08 (0.98)	0.07 (0.98)	0.06 (0.96)	--
Age-adjusted	Ref.	0.04 (-0.02, 0.10)	0.10 (0.04, 0.15)	0.08 (0.03, 0.14)	0.06 (0.003, 0.11)	0.02
Multivariable-adjusted[d]	Ref.	0.03 (-0.03, 0.08)	0.08 (0.02, 0.13)	0.06 (0.002, 0.11)	0.03 (-0.03, 0.09)	0.21
**Global cognition[c]**						
Standardized score mean (SD)	-0.05 (1.02)	0.01 (0.98)	0.05 (0.98)	0.03 (1.00)	0.04 (0.98)	--
Age-adjusted	Ref.	0.03 (-0.02, 0.09)	0.07 (0.01, 0.12)	0.04 (-0.01, 0.10)	0.03 (-0.02, 0.09)	0.27
Multivariable-adjusted[d]	Ref.	0.02 (-0.03, 0.08)	0.05 (-0.005, 0.11)	0.02 (-0.04, 0.08)	0.01 (-0.05, 0.07)	0.88

[a] Energy-adjusted gluten intake was cumulatively averaged from 1991 to the last questionnaire cycle preceding the cognitive assessment.

[b] *P*-trend was calculated using the median gluten intake for each quintile as a continuous variable.

[c] Standardized scores for psychomotor speed/attention, learning/working memory, and global cognition were calculated by standardizing as the mean of the standardized scores of the following CogState battery tasks: Detection + Identification, One Card Learning + One-Back, and all four tasks, respectively. Higher scores indicate better performance, with one unit increase represents 1 SD higher than the mean.

[d] Model was adjusted for age (years), race (white, non-white), body mass index (kg/m^2), husband's educational attainment (high school or below, college, graduate school, unmarried/missing), family income (dollar), history of diabetes (yes, no), history of hypertension (yes, no), history of hypercholesterolemia (yes, no), history of myocardial infarction (yes, no), current depression status (yes, no), smoking (never, past, current, missing), aspirin or non-steroid anti-inflammatory drug use (yes, no), multivitamins use (yes, no), physical activity (metabolic equivalents/week), menopausal status and hormone use (pre-menopause, post-menopause and never/past/current user of hormone therapy), total energy intake (kcal/d), alcohol intake (g/d), and Alternative Healthy Eating Index score (excluding alcohol and whole grains).

© 2021 Wang Y et al. *JAMA Network Open.*

eTable 5. Sensitivity Analysis of the Mean Differences in Standardized Cognitive Scores Associated With Quintiles of Gluten Intake Among 13 486 women Who Had Never Reported Dementia Diagnosis[a]

Model	Quintiles of average gluten intake					*P*-trend[b]
	Q1 (lowest)	Q2	Q3	Q4	Q5 (Highest)	
**Psychomotor speed/attentionv[c]**						
Standardized score mean (SD)	-0.03 (1.03)	0.01 (1.00)	0.02 (0.96)	-0.002 (1.02)	0.02 (0.99)	--
Age-adjusted	Ref.	0.02 (-.04, 0.07)	0.02 (-.03, 0.07)	-0.01 (-0.07, 0.04)	-0.005 (-0.06, 0.05)	0.53
Multivariable-adjusted[d]	Ref.	0.01 (-0.04, 0.06)	0.01 (-0.04, 0.07)	-0.02 (-0.08, 0.03)	-0.02 (-0.07, 0.03)	0.21
**Learning/working memory[c]**						
Standardized score mean (SD)	-0.04 (0.98)	0.001 (0.96)	0.07 (0.98)	0.05 (0.98)	0.05 (0.96)	--
Age-adjusted	Ref.	0.03 (-0.02, 0.08)	0.09 (0.04, 0.14)	0.06 (0.01, 0.11)	0.05 (-0.003, 0.10)	0.04
Multivariable-adjusted[d]	Ref.	0.01 (-0.04, 0.07)	0.07 (0.02, 0.13)	0.04 (-0.01, 0.09)	0.02 (-0.03, 0.07)	0.30
**Global cognition[c]**						
Standardized score mean (SD)	-0.05 (1.02)	-0.0002 (0.98)	0.05 (0.97)	0.02 (1.02)	0.03 (0.97)	--
Age-adjusted	Ref.	0.02 (-0.03, 0.08)	0.06 (0.01, 0.12)	0.02 (-0.03, 0.07)	0.02 (-0.03, 0.07)	0.50
Multivariable-adjusted[d]	Ref.	0.01 (-0.04, 0.07)	0.05 (-0.003, 0.10)	0.01 (-0.05, 0.06)	-0.002 (-0.05, 0.05)	0.77

[a] Energy-adjusted gluten intake was cumulatively averaged from 1991 to the last questionnaire cycle preceding the cognitive assessment.

[b] *P*-trend was calculated using the median gluten intake for each quintile as a continuous variable.

[c] Standardized scores for psychomotor speed/attention, learning/working memory, and global cognition were calculated by standardizing as the mean of the standardized scores of the following CogState battery tasks: Detection + Identification, One Card Learning + One-Back, and all four tasks, respectively. Higher scores indicate better performance, with one unit increase represents 1 SD higher than the mean.

[d] Model was adjusted for age (years), race (white, non-white), body mass index (kg/m^2), husband's educational attainment (high school or below, college, graduate school, unmarried/missing), family income (dollar), history of diabetes (yes, no), history of hypertension (yes, no), history of hypercholesterolemia (yes, no), history of myocardial infarction (yes, no), current depression status (yes, no), smoking (never, past, current, missing), aspirin or non-steroid anti-inflammatory drug use (yes, no), multivitamins use (yes, no), physical activity (metabolic equivalents/week), menopausal status and hormone use (pre-menopause, post-menopause and never/past/current user of hormone therapy), total energy intake (kcal/d), alcohol intake (g/d), and Alternative Healthy Eating Index score (excluding alcohol and whole grains).

© 2021 Wang Y et al. *JAMA Network Open.*

eTable 6. Sensitivity Analysis of the Mean Differences in Standardized Cognitive Scores Associated With Quintiles of Gluten Intake Among 11 646 Women Who Completed All Food Frequency Questionnaires During 24-Year Follow-up Prior to Cognitive Assessment[a]

Model	Quintiles of average gluten intake					*P*-trend[b]
	Q1 (lowest)	Q2	Q3	Q4	Q5 (Highest)	
**Psychomotor speed/attentionv[c]**						
Standardized score mean (SD)	-0.005 (0.97)	0.001 (1.00)	0.02 (0.95)	-0.01 (1.01)	0.03 (0.99)	--
Age-adjusted	Ref.	-0.01 (-0.07, 0.04)	-0.002 (-0.06, 0.05)	-0.05 (-0.10, 0.01)	-0.02 (-0.08, 0.04)	0.33
Multivariable-adjusted[d]	Ref.	-0.01 (-0.07, 0.04)	-0.004 (-0.06, 0.05)	-0.05 (-0.11, 0.01)	-0.03 (-0.09, 0.03)	0.18
**Learning/working memory[c]**						
Standardized score mean (SD)	-0.04 (0.97)	-0.01 (0.95)	0.09 (0.97)	0.05 (0.98)	0.06 (0.97)	--
Age-adjusted	Ref.	0.01 (-0.05, 0.07)	0.10 (0.04, 0.16)	0.05 (-0.004, 0.11)	0.05 (-0.01, 0.11)	0.04
Multivariable-adjusted[d]	Ref.	0.0004 (-0.06, 0.06)	0.09 (0.03, 0.14)	0.04 (-0.02, 0.09)	0.03 (-0.03, 0.09)	0.20
**Global cognition[c]**						
Standardized score mean (SD)	-0.03 (1.01)	-0.01 (0.98)	0.05 (0.96)	0.02 (1.02)	0.05 (0.98)	--
Age-adjusted	Ref.	-0.004 (-0.06, 0.05)	0.05 (-0.003, 0.11)	-0.002 (-0.06, 0.05)	0.01 (-0.04, 0.07)	0.67
Multivariable-adjusted[d]	Ref.	-0.01 (-0.07, 0.05)	0.04 (-0.01, 0.10)	-0.01 (-0.07, 0.04)	-0.003 (-0.06, 0.05)	0.83

[a] Energy-adjusted gluten intake was cumulatively averaged from 1991 to the last questionnaire cycle preceding the cognitive assessment.

[b] *P*-trend was calculated using the median gluten intake for each quintile as a continuous variable.

[c] Standardized scores for psychomotor speed/attention, learning/working memory, and global cognition were calculated by standardizing as the mean of the standardized scores of the following CogState battery tasks: Detection + Identification, One Card Learning + One-Back, and all four tasks, respectively. Higher scores indicate better performance, with one unit increase represents 1 SD higher than the mean.

[d] Model was adjusted for age (years), race (white, non-white), body mass index (kg/m^2), husband's educational attainment (high school or below, college, graduate school, unmarried/missing), family income (dollar), history of diabetes (yes, no), history of hypertension (yes, no), history of hypercholesterolemia (yes, no), history of myocardial infarction (yes, no), current depression status (yes, no), smoking (never, past, current, missing), aspirin or non-steroid anti-inflammatory drug use (yes, no), multivitamins use (yes, no), physical activity (metabolic equivalents/week), menopausal status and hormone use (pre-menopause, post-menopause and never/past/current user of hormone therapy), total energy intake (kcal/d), alcohol intake (g/d), and Alternative Healthy Eating Index score (excluding alcohol and whole grains).

© 2021 Wang Y et al. *JAMA Network Open.*

eTable 7. Multivariable-Adjusted Mean Differences in Standardized Cognitive Scores Associated With Quintiles of Gluten Intake by 4-Year Interval Prior to Cognitive Assessment Among 11 646 Women[a]

Years prior to cognitive assessment	Quintiles of gluten intake					*P*-trend[b]
	Q1 (lowest)	Q2	Q3	Q4	Q5 (highest)	
**Psychomotor speed/attention[c]**						
20-24 years	Ref.	-0.01 (-0.07, 0.04)	-0.02 (-0.07, 0.04)	-0.02 (-0.07, 0.04)	-0.01 (-0.07, 0.04)	0.66
16-20 years	Ref.	0.003 (-0.05, 0.06)	0.005 (-0.05, 0.06)	-0.02 (-0.08, 0.04)	-0.01 (-0.07, 0.05)	0.53
12-16 years	Ref.	-0.03 (-0.08, 0.03)	0.01 (-0.05, 0.06)	-0.04 (-0.10, 0.01)	-0.03 (-0.09, 0.02)	0.22
8-12 years	Ref.	-0.02 (-0.08, 0.04)	-0.04 (-0.10, 0.02)	-0.03 (-0.08, 0.03)	-0.04 (-0.09, 0.02)	0.23
4-8 years	Ref.	-0.04 (-0.10, 0.02)	-0.06 (-0.12, -0.001)	-0.06 (-0.11, 0.0002)	-0.03 (-0.09, 0.03)	0.35
≤4 years	Ref.	0.01 (-0.04, 0.07)	0.01 (-0.05, 0.06)	0.02 (-0.03, 0.08)	-0.01 (-0.07, 0.04)	0.66
Distant past (12-24 years)§	Ref.	0.03 (-0.03, 0.09)	0.002 (-0.06, 0.06)	-0.01 (-0.07, 0.05)	-0.02 (-0.08, 0.05)	0.28
Recent past (4-12 years)[d]	Ref.	-0.04 (-0.10, 0.02)	-0.03 (-0.09, 0.03)	-0.04 (-0.10, 0.02)	-0.02 (-0.09, 0.04)	0.56
**Learning/working memory[c]**						
20-24 years	Ref.	0.03 (-0.03, 0.08)	0.05 (-0.01, 0.11)	0.06 (0.00, 0.11)	0.03 (-0.02, 0.09)	0.21
16-20 years	Ref.	0.04 (-0.02, 0.10)	0.02 (-0.04, 0.07)	0.04 (-0.02, 0.09)	0.02 (-0.04, 0.07)	0.71
12-16 years	Ref.	0.07 (0.02, 0.13)	0.10 (0.04, 0.15)	0.09 (0.03, 0.14)	0.05 (-0.00, 0.11)	0.11
8-12 years	Ref.	0.02 (-0.03, 0.08)	0.03 (-0.02, 0.09)	0.02 (-0.03, 0.08)	0.01 (-0.05, 0.07)	0.79
4-8 years	Ref.	-0.003 (-0.06, 0.05)	-0.01 (-0.06, 0.05)	0.02 (-0.04, 0.07)	0.02 (-0.04, 0.07)	0.38
≤4 years	Ref.	0.05 (-0.01, 0.10)	0.04 (-0.01, 0.10)	0.05 (-0.01, 0.11)	0.04 (-0.02, 0.09)	0.28
Distant past (12-24 years)[d]	Ref.	0.03 (-0.02, 0.09)	0.05 (-0.01, 0.11)	0.02 (-0.04, 0.08)	0.03 (-0.03, 0.09)	0.50
Recent past (4-12 years)[d]	Ref.	0.002 (-0.05, 0.06)	0.04 (-0.02, 0.10)	0.02 (-0.03, 0.08)	0.01 (-0.06, 0.07)	0.70
**Global cognition[c]**						
20-24 years	Ref.	0.01 (-0.05, 0.06)	0.02 (-0.04, 0.07)	0.02 (-0.04, 0.08)	0.01 (-0.05, 0.07)	0.71
16-20 years	Ref.	0.02 (-0.03, 0.08)	0.01 (-0.04, 0.07)	0.01 (-0.05, 0.06)	0.002 (-0.05, 0.06)	0.83
12-16 years	Ref.	0.02 (-0.03, 0.08)	0.06 (-0.00, 0.11)	0.02 (-0.04, 0.07)	0.01 (-0.05, 0.06)	0.98
8-12 years	Ref.	-0.002 (-0.06, 0.05)	-0.01 (-0.07, 0.05)	-0.01 (-0.06, 0.05)	-0.02 (-0.08, 0.04)	0.51
4-8 years	Ref.	-0.03 (-0.08, 0.03)	-0.04 (-0.10, 0.02)	-0.03 (-0.09, 0.03)	-0.01 (-0.07, 0.05)	0.88
≤4 years	Ref.	0.04 (-0.02, 0.09)	0.03 (-0.03, 0.08)	0.04 (-0.01, 0.10)	0.01 (-0.05, 0.07)	0.78
Distant past (12-24 years)[d]	Ref.	0.04 (-0.02, 0.10)	0.03 (-0.03, 0.08)	0.002 (-0.06, 0.06)	0.01 (-0.06, 0.07)	0.69
Recent past (4-12 years)[d]	Ref.	-0.03 (-0.08, 0.03)	0.0001 (-0.06, 0.06)	-0.02 (-0.08, 0.04)	-0.01 (-0.08, 0.05)	0.85

[a] Energy-adjusted gluten intake was derived from individual assessment conducted in the respective year category, except for distant past and recent past intakes, which were cumulative averaged through 12-24 years and 4-12 years prior to cognitive assessment., respectively. Model was adjusted for age (years), race (white, non-white), body mass index (kg/m^2), husband's educational attainment (high school or below, college, graduate school, unmarried/missing), family income (dollar), history of diabetes (yes, no), history of hypertension (yes, no), history of hypercholesterolemia (yes, no), history of myocardial infarction (yes, no), current depression status (yes, no), smoking (never, past, current, missing), aspirin or non-steroid anti-inflammatory drug use (yes, no), multivitamins use (yes, no), physical activity (metabolic equivalents/week), menopausal status and hormone use (pre-menopause, post-menopause and never/past/current user of hormone therapy), total energy intake (kcal/d), alcohol intake (g/d), and Alternative Healthy Eating Index score (excluding alcohol and whole grains).

[b] *P*-trend was calculated using the median gluten intake for each quintile as a continuous variable

[c] Standardized scores for psychomotor speed/attention, learning/working memory, and global cognition were calculated by standardizing as the mean of the standardized scores of the following CogState battery tasks: Detection + Identification, One Card Learning + One-Back, and all four tasks, respectively. Higher scores indicate better performance, with one unit increase represents 1 SD higher than the mean.

[d] Distant and recent past intakes were mutually adjusted.

© 2021 Wang Y et al. *JAMA Network Open.*

eTable 8. Multivariable-Adjusted Mean Differences in Standardized Cognitive Scores Associated With 1 SD (1.98 g/d) Increase in Mean Gluten Intake From Distant Past (12-24 y) to Recent Past (4-12 y) of Cognitive Assessment Among 11 646 Women[a]

Composite cognitive scores[b]	Estimate (95% confidence interval)	*P*
Psychomotor speed/attention	-0.01 (-0.03, 0.01)	0.32
Learning/working memory	-0.003 (-0.02, 0.02)	0.75
Global cognition	-0.01 (-0.02, 0.01)	0.62

[a] Energy-adjusted gluten intake was cumulatively averaged from 1991 to the last questionnaire cycle preceding the cognitive assessment. Model was adjusted for age (years), race (white, non-white), body mass index (kg/m^2), husband's educational attainment (high school or below, college, graduate school, unmarried/missing), family income (dollar), history of diabetes (yes, no), history of hypertension (yes, no), history of hypercholesterolemia (yes, no), history of myocardial infarction (yes, no), current depression status (yes, no), smoking (never, past, current, missing), aspirin or non-steroid anti-inflammatory drug use (yes, no), multivitamins use (yes, no), physical activity (metabolic equivalents/week), menopausal status and hormone use (pre-menopause, post-menopause and never/past/current user of hormone therapy), total energy intake (kcal/d), alcohol intake (g/d), and Alternative Healthy Eating Index score (excluding alcohol and whole grains).

[b] Standardized scores for psychomotor speed/attention, learning/working memory, and global cognition were calculated by standardizing as the mean of the standardized scores of the following CogState battery tasks: Detection + Identification, One Card Learning + One-Back, and all four tasks, respectively. Higher scores indicate better performance, with one unit increase represents 1 SD higher than the mean.

© 2021 Wang Y et al. *JAMA Network Open.*

eFigure 1. Nurses' Health Study II Questionnaire in 2015 Questionnaire Cycle

NURSES' HEALTH STUDY II

HARVARD
T.H. CHAN
SCHOOL OF PUBLIC HEALTH

Channing Laboratory • 181 Longwood Avenue • Boston, Massachusetts 02115-5804

Telephone (617) 525-2279
• Fax (617) 525-2008
• E-Mail NHS2@channing.harvard.edu

This is your ID ➡

3/8" PERF

Your email:
Please print your email address in the box so that we may send you occasional updates from the Nurses' Health Study II.

Please print neatly and differentiate numbers and letters (e.g., 1 vs l or i, Ø vs O, 5 vs S)

We will not release your e-mail address to anyone!

© 2021 Wang Y et al. *JAMA Network Open.*

INSTRUCTIONS

INTERNET:
Go to our website at www.NHS2.org and use your ID number (see front of this page) and your birth date to log in. Follow the instructions on the screen to complete the survey online.

PAPER FORM:
Please use an ordinary No. 2 pencil to answer all questions. Fill in the appropriate response circles completely. The form is designed to be read by optical-scanning equipment, so it is important that you keep any write-in responses within the spaces provided and erase any incorrect marks completely. If you have comments, please write them on a separate piece of paper.

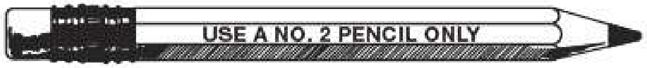

EXAMPLE A) Mark "Yes" bubble and Year of Diagnosis bubble for each illness you have had diagnosed.

19. Since June 2013, have you had any of these clinician-diagnosed illnesses?

LEAVE BLANK FOR "NO", MARK HERE FOR "YES"

		YEAR OF DIAGNOSIS: Before June 1 2013	June '13 to May '15	After June 1 2015
Myocardial infarction (heart attack)	Ⓨ	○	○	○
Angina pectoris	●	○	●	○
Confirmed by angiography? ○ No ● Yes				
Coronary bypass, angioplasty or stent	●	○	○	●
Transient ischemic attack (TIA)	Ⓨ	○	○	○

Please fill in the circles completely; do not mark this way: ✓ ✗ ◒

EXAMPLE B) AGE natural periods ceased:

AGE	
4	6

Print numbers neatly within boxes. Your writing will be read by our scanner.

- **Please tear off the cover letter (to preserve confidentiality) and return the questionnaire in the enclosed postage–paid envelope.**
- **If your name and address as printed on this questionnaire are no longer correct or are incomplete, or if you are providing your email address, please make any necessary changes on the letter and return it to us.**
- **Thank you for completing the 2015 Nurses' Health Study II Questionnaire.**

Federal research regulations require us to include the following information:
There are no direct benefits to you from participating in this study.
The risk of breach of confidentiality associated with participation in this study is very small.
Your choice to participate in this study is completely voluntary and you may decline or withdraw at any time without penalty.
You may skip any question you do not wish to answer.
You will not receive monetary compensation for participating.
If you have any questions regarding your rights as a research participant, you are encouraged to call a representative of the Human Subjects Committee at the Brigham and Women's Hospital (617-424-4100).

© 2021 Wang Y et al. *JAMA Network Open.*

1. PLEASE USE PENCIL

CURRENT WEIGHT

POUNDS

2. Do you currently smoke cigarettes?
No / Yes → How many/day? 1–4 / 5–14 / 15–24 / 25–34 / 35–44 / 45+

3. Have you had your uterus removed?
No / Yes → Date of surgery: Before June 1, 2013 / After June 1, 2013

4. Have you ever had either of your ovaries surgically removed?
No / Yes → a) How many ovaries do you have remaining? None / One

5. Have your natural menstrual periods ceased PERMANENTLY?
- No: Premenopausal
- Yes: No menstrual periods
- Yes: Had menopause but now have periods induced by hormones
- Not sure (e.g., perimenopausal)

a) AGE natural periods ceased: AGE

b) For what reason did your periods cease? Natural / Surgery / Endometrial ablation / Radiation or Chemo.

6. Are you currently using oral contraceptives for any reason?
No / Yes

7. Since June 2013, have you used prescription female hormones? (Not including oral contraceptives.)
Yes → a) How many months did you use hormones since June 2013?
1–4 months / 5–9 / 10–14 / 15–19 / 20–25 / 26–30 / 31–35 / 36+ months
No

b) Are you *currently* using them (within the last month)? Yes / No If No, skip to Part d

c) Mark the type(s) of hormones you are *CURRENTLY* using:
- Combined: Prempro / Premphase / Combipatch / FemHRT
- Estrogen: Oral Premarin or conjugated estrogen / Patch Estrogen / Vaginal Estrogen / Estrace / Estrogen gels, creams, or sprays on skin / Estratest / Other Estrogen (specify in box below)
- Progesterone/Progestin: Provera/Cycrin/MPA / Vaginal / Micronized (e.g., Prometrium) / Other progesterone (specify type)
- Other hormones *CURRENTLY* used: Bioidentical estrogen / Testosterone / Bioidentical progesterone / Other (specify in box)

d) If you used oral conjugated estrogen (e.g., Premarin) since June 2013, what dose did you usually take?
.30 mg/day or less / .45 mg/day / .625 mg/day / .9 mg/day / 1.25 mg/day or higher / Unsure / Did not take oral conjugated estrogen

8. Are you currently using any of these over-the-counter (OTC) preparations for hormone replacement?
Soy estrogen products / Black cohosh (e.g., Remifemin) / Natural progesterone cream/wild yam / Other OTC

9. In the past two years, have you had two weeks or longer when nearly every day you felt sad, blue or depressed for most of the day?
No / Yes

10. In the past two years, have you had gastrointestinal bleeding that required hospitalization or a transfusion?
No / Yes

11. In the past year, have you been bothered by constipation for at least 12 weeks (not necessarily consecutive)?
No / Yes → If Yes: I have seen my primary care physician / I have seen a specialist (e.g., gastroenterologist) / Neither

12. Has your spouse (or sleep partner) ever told you that you appear to "act out your dreams" while sleeping (punched or flailed arms in the air, shouted or screamed), on three or more occasions?
No / Yes / I do not have a sleep partner

13. Please estimate an average of the time when you fall asleep and wake up, over the past 2 years on WORK-FREE DAYS, when you were without obligations and not using an alarm clock to wake up:

I usually fall asleep at ___ hour ___ minute AM / PM (This is NOT when you get into bed!)

I usually wake up at ___ hour ___ minute AM / PM (This may NOT be when you get OUT of bed!)

I always use alarm clock to wake up on free days

14. What proportion of the dairy products and fruit & vegetables you consume are organic?

Dairy products:	None	Less than 5%	5–10%	11–25%	26–50%	51–75%	75%+
Fruit & Vegetables:	None	Less than 5%	5–10%	11–25%	26–50%	51–75%	75%+

15. How often do you eat canned food? (e.g., vegetables, tuna, soup, tomato sauce, etc., in metal cans)
<1 per month / 1 to 3 per month / 1 to 4 per week / 5 to 7 per week / 2 per day / 3+ per day

16. How often do you drink canned beverages? (e.g., soda, beer, juices, etc., in metal cans)
<1 per month / 1 to 3 per month / 1 to 4 per week / 5 to 7 per week / 2 per day / 3+ per day

17. Has a clinician ever diagnosed you with any of the following conditions?
Celiac disease / Fatty liver disease and/or cirrhosis / Glaucoma / Sarcoidosis / None of these

18. In the past two years, have you been diagnosed with an episode of:

a) Diverticulitis (NOT diverticulosis) that required antibiotics and/or hospitalization?
No / Yes → If Yes, did you require surgery for diverticulitis? No / Yes

b) Diverticular bleeding that required blood transfusion and/or hospitalization? No / Yes

c) Diverticulosis of the colon WITHOUT diverticulitis or diverticular bleeding? No / Yes

© 2021 Wang Y et al. *JAMA Network Open.*

19. Since June 2013, have you had any of these clinician-diagnosed illnesses?

LEAVE BLANK FOR "NO", MARK HERE FOR "YES"

Illness	Yes	YEAR OF DIAGNOSIS: Before June 1 2013	June '13 to May '15	After June 1 2015
Myocardial infarction (heart attack)	Y			
Angina pectoris	Y			
Confirmed by angiography? No / Yes				
Coronary bypass, angioplasty or stent	Y			
Transient ischemic attack (TIA)	Y			
Stroke (CVA)	Y			
Pulmonary embolus	Y			
Deep vein thrombosis	Y			
Fibrocystic/other benign breast disease	Y			
Confirmed by breast biopsy? No / Yes				
Breast cancer	Y			
Cancer of the uterus (endometrium)	Y			
Cancer of the ovary	Y			
Colon or rectal polyp (benign)	Y			
Cancer of the colon or rectum	Y			
Melanoma	Y			
Basal cell skin cancer	Y			
Squamous cell skin cancer	Y			
Other cancer	Y			
Specify site of other cancer				
Crohn's/Ulcerative or Microscopic colitis	Y			
Dysplastic nevus (Atypical mole)	Y			
Gastric or duodenal ulcer	Y			
Barrett's esophagus	Y			
Cholecystectomy	Y			
Diabetes mellitus	Y			
Elevated cholesterol	Y			
High blood pressure	Y			
Kidney stones	Y			
Multiple Sclerosis	Y			
Asthma, Clinician diagnosed	Y			
Emphysema/Chronic bronchitis	Y			
Graves' disease/Hyperthyroidism	Y			
Hypothyroidism	Y			
Hyperparathyroidism	Y			
Gout	Y			
Peripheral neuropathy	Y			
SLE (systemic lupus)	Y			
Rheumatoid arthritis	Y			
Other arthritis	Y			
Depression, Clinician diagnosed	Y			
Parkinson's disease	Y			
Alzheimer's or other dementia	Y			
Gastric banding/bypass/sleeve	Y			
Fractures: Wrist or Colles' Fracture	Y			
Hip Fracture	Y			
Vertebral fracture	Y			
Other major illness or surgery since June 2013	Y			

Please specify: Date:

FOR OFFICE USE ONLY

20. In the past two years have you had:

(If yes, mark all that apply)

	No	Yes, for screening	Yes, for symptoms
A physical exam?	N	Y	Y
Mammogram (or other breast imaging)?	N	Y	Y
Eye exam by doctor?	N	Y	Y
Fasting blood sugar?	N	Y	Y

- Upper endoscopy? No / Yes
- (Virtual) CT Colonoscopy? No / Yes
- Colonoscopy? No / Yes
- Sigmoidoscopy? No / Yes

Initial reason(s) you had Colonoscopy/Sigmoidoscopy?

- Visible blood
- Occult fecal blood
- Abdominal pain
- Diarrhea/constipation
- Family history of colon cancer
- Fecal/stool DNA testing
- Follow-up of (virtual) CT colonoscopy
- Prior polyps
- Asymptomatic or routine screening

21. Regular Medication *(Mark if used regularly in past 2 years.)*

- Acetaminophen (e.g., Tylenol)
  - **Days/week:** 1 / 2–3 / 4–5 / 6+ days
  - **Tablets/wk:** 1–2 / 3–5 / 6–14 / 15+ tablets
- "Baby" or low dose aspirin (100 mg or less/tablet)
  - **Days/week:** 1 / 2–3 / 4–5 / 6+ days
  - **Tablets/wk:** 1–2 / 3–5 / 6–14 / 15+ tablets
- Aspirin or aspirin-containing products (325 mg or more/tablet)
  - **Days/week:** 1 / 2–3 / 4–5 / 6+ days
  - **Tablets/wk:** 1–2 / 3–5 / 6–14 / 15+ tablets
- Ibuprofen (e.g., Advil, Motrin, Nuprin)
  - **Days/week:** 1 / 2–3 / 4–5 / 6+ days
  - **Tablets/wk:** 1–2 / 3–5 / 6–14 / 15+ tablets
- Celebrex (COX-2 inhibitors)
  - **Days/week:** 1 / 2–3 / 4–5 / 6+ days
- Other anti-inflammatory analgesics, 2+ times/week (e.g., Aleve, Clinoril, Relafen, Indocin)
- Thiazide diuretic / Lasix / Potassium
- Calcium blocker (e.g., Calan, Procardia, Cardizem, Norvasc)
- Beta-blocker (e.g., Lopressor, Tenormin, Corgard, Coreg)
- ACE inhibitors (e.g., Capoten, Vasotec, Zestril)
- Angiotensin receptor blocker (e.g., Diovan, Cozaar, Avapro)
- Other anti-hypertensive (e.g., clonidine, doxazosin)
- Coumadin / Pradaxa/Xarelto/Eliquis / Plavix
- Prasugrel (Effient) / Digoxin / Antiarrhythmic
- Mevacor (lovastatin) / Zocor (simvastatin) / Crestor
- Pravachol (pravastatin) / Lipitor (atorvastatin) / Other statin
- Other cholesterol-lowering drug [e.g., niacin, Lopid (gemfibrozil), Tricor (fenofibrate), Questran (cholestyramine), Colestid, Zetia]
- Steroids taken orally (e.g., Prednisone, Decadron, Medrol)
- Insulin / Metformin / Other oral hypoglycemic agents
- Thyroid hormone (e.g., Synthroid, Levothroid, Levoxyl)
- Triptans (e.g., Imitrex, Maxalt, Zomig, Amerge, Replax)
- Bisphosphonates (e.g., Fosamax, Boniva, Actonel)
- Evista (raloxifene) / Nolvadex (tamoxifen)
- Anticholinergics (e.g., Detrol, Ditropan, Vesicare)
- SSRIs (e.g., Celexa, Lexapro, Prozac, Paxil, Zoloft, Luvox)
- Tricyclics (e.g., amitriptyline, nortriptyline, imipramine)
- Other antidepressants (e.g., Wellbutrin, Effexor, Remeron)
- Minor tranquilizers (e.g., Valium, alprazolam, lorazepam)
- Prilosec, Nexium, Prevacid, Protonix, Aciphex, Dexilant
- H2 blocker (e.g., Pepcid, Tagamet, Zantac, Axid)

22. Is this your correct date of birth?

- Yes
- No → If no, please write correct date. MONTH / DAY / YEAR

3/8" PERF

© 2021 Wang Y et al. *JAMA Network Open.*

23. Do you currently take multivitamins? (Please report other individual vitamins in the next section.)

○ Yes → a) How many do you take per week? ○ 2 or less ○ 3–5 ○ 6–9 ○ 10 or more

○ No b) What specific brand (or equivalency) do you usually take?

○ Centrum Silver or Senior vit. ○ Centrum or generic equiv. ○ Any Multi-vit. Pack

○ Eye Vitamin, e.g., Ocuvite ○ One-A-Day Women's ○ Other →

Not counting multivitamins, do you take any of the following vitamin preparations?

a) Vitamin A	○ No	○ Yes, seasonal only / ○ Yes, most months	If Yes, Dose per day:	○ Less than 10,000 IU	○ 10,000 to 15,000 IU	○ 16,000 to 22,000 IU	○ 23,000 IU or more	○ Don't know
b) Potassium	○ No	○ Yes	If Yes, Dose per day:	○ Less than 2.5 mEq (100 mg)	○ 3 to 9 mEq	○ 10 to 19 mEq	○ 20 mEq or more	○ Don't know
c) Vitamin C	○ No	○ Yes, seasonal only / ○ Yes, most months	If Yes, Dose per day:	○ Less than 400mg	○ 400 to 700 mg	○ 750 to 1250 mg	○ 1300 mg or more	○ Don't know
d) Vitamin B_6	○ No	○ Yes	If Yes, Dose per day:	○ Less than 50 mg	○ 50 to 99 mg	○ 100 to 149 mg	○ 150 mg or more	○ Don't know
e) Vitamin E	○ No	○ Yes	If Yes, Dose per day:	○ Less than 100IU	○ 100 to 250 IU	○ 300 to 500 IU	○ 600 IU or more	○ Don't know
			Type: ○ Natural	○ Regular (dl)	○ Unknown			
f) Calcium (Include Calcium in Tums, etc.)	○ No	○ Yes	If Yes, Dose per day: (elemental calcium)	○ Less than 600 mg	○ 600 to 900 mg	○ 901 to 1500 mg	○ 1501 mg or more	○ Don't know
g) Selenium	○ No	○ Yes	If Yes, Dose per day:	○ Less than 60 mcg	○ 80 to 130 mcg	○ 140 to 250 mcg	○ 260 mcg or more	○ Don't know
h) Vitamin D (in calcium supplement or separately)	○ No	○ Yes, seasonal only / ○ Yes, most months	If Yes, Dose per day:	○ Less than 600 IU	○ 600 to 900 IU	○ 1000 to 1900 IU	○ 2000 IU or more	○ Don't know
i) Zinc	○ No	○ Yes	If Yes, Dose per day:	○ Less than 31 mg	○ 31 to 74 mg	○ 75 to 100 mg	○ 101 mg or more	○ Don't know

24. Are there other supplements that you take on a regular basis?

○ Metamucil/Citrucel ○ Beta-carotene ○ Chromium ○ Folic Acid ○ Glucosamine/Chondroitin
○ Cod Liver Oil ○ Probiotics ○ Vitamin Water ○ B-Complex ○ Iron ○ Magnesium
○ Fish Oil ○ Melatonin ○ Coenzyme Q10 ○ Ginkgo Biloba
○ Flax Seed Oil ○ Vitamin B12 ○ Niacin ○ Lycopene ○ Other →

25. How many teaspoons of sugar do you add to your beverages or food each day?

○ Zero ○ 1 tsp. ○ 2 tsp. ○ 3 tsp. ○ 4 tsp. ○ 5 tsp.
○ 6 tsp. ○ 7 tsp. ○ 8 tsp. ○ 9 tsp. ○ 10 tsp.
More than 10? Write number here → ____ tsp.

26. What brand and type of cold breakfast cereal do you usually eat? → Specify cereal brand & type (e.g., Kellogg's Raisin Bran)

○ Don't eat cold breakfast cereal.

27. What form of margarine or spread do you usually use (exclude pure butter?) → What specific brand & type of margarine (e.g., Shedd's Country Crock plus calcium and vitamins)

○ None Form? ○ Stick ○ Tub ○ Spray ○ Squeeze (liquid)

Type? ○ Reg ○ Light ○ Nonfat

28. For each food listed, fill in the circle indicating how often on average you have used the amount specified during the past year.

AVERAGE USE LAST YEAR

DAIRY FOODS		Never, or less than once per month	1–3 per month	1 per week	2–4 per week	5–6 per week	1 per day	2–3 per day	4–5 per day	6+ per day
Milk (8 oz. glass)	Skim milk	○	○	Ⓦ	○	○	Ⓓ	○	○	○
	1 or 2 % milk	○	○	Ⓦ	○	○	Ⓓ	○	○	○
	Whole milk	○	○	Ⓦ	○	○	Ⓓ	○	○	○
	Soy milk	○	○	Ⓦ	○	○	Ⓓ	○	○	○
Cream, e.g., coffee, sour (exclude fat free) (1 Tbs)		○	○	Ⓦ	○	○	Ⓓ	○	○	○
Non-dairy coffee whitener (exclude fat free) (1 Tbs)		○	○	Ⓦ	○	○	Ⓓ	○	○	○
Frozen yogurt, sherbet, sorbet or low-fat ice cream (1 c)		○	○	Ⓦ	○	○	Ⓓ	○	○	○
Regular ice cream (1 cup)		○	○	Ⓦ	○	○	Ⓓ	○	○	○
Spreads added to food or bread; exclude use in cooking	Pure butter	○	○	Ⓦ	○	○	Ⓓ	○	○	○
	Margarine (e.g., Country Crock)	○	○	Ⓦ	○	○	Ⓓ	○	○	○
	"Spreadable Butter"- butter/oil blend (e.g., Olivio Spread But.)	○	○	Ⓦ	○	○	Ⓓ	○	○	○
Yogurt (4–6 oz.)	Plain	○	○	Ⓦ	○	○	Ⓓ	○	○	○
	Artificially sweetened (e.g., light peach)	○	○	Ⓦ	○	○	Ⓓ	○	○	○
	Sweetened (e.g., strawberry, vanilla)	○	○	Ⓦ	○	○	Ⓓ	○	○	○
Cottage or ricotta cheese (1/2 cup)		○	○	Ⓦ	○	○	Ⓓ	○	○	○
Cream cheese (1 oz.)		○	○	Ⓦ	○	○	Ⓓ	○	○	○
Other cheese, e.g., American, cheddar, etc., plain or as part of a dish (1 slice or 1 oz. serving)		○	○	Ⓦ	○	○	Ⓓ	○	○	○

→ What type of cheese do you usually eat? ○ Regular ○ Low-fat or Lite ○ Nonfat ○ None

© 2021 Wang Y et al. *JAMA Network Open.*

28. *(continued)* **For each food listed, fill in the circle indicating how often on average you have used the amount specified during the past year.**

Please try to average your seasonal use of foods over the entire year. For example, if a food such as cantaloupe is eaten 4 times a week during the approximate 3 months that it is in season, then the average use would be once per week.

FRUITS	Never, or less than once per month	1-3 per month	1 per week	2-4 per week	5-6 per week	1 per day	2-3 per day	4-5 per day	6+ per day
Raisins (1 oz. or small pack) or grapes (1/2 cup)			W			D			
Prunes or dried plums (1/2 cup canned or 1/4 cup dried)			W			D			
Bananas (1)			W			D			
Cantaloupe (1/4 melon)			W			D			
Avocado (1/2 fruit or 1/2 cup)			W			D			
Fresh apples or pears (1)			W			D			
Apple juice or cider (small glass)			W			D			
Tangerines, clementines, mandarin oranges (1)			W			D			
Oranges (1)			W			D			
Orange juice (small glass): Calcium or Vit. D fortified			W			D			
Orange juice (small glass): Regular (not calcium fortified)			W			D			
Grapefruit (1/2)			W			D			
Grapefruit juice (small glass)			W			D			
Other fruit juices (e.g., cranberry, grape) (small glass)			W			D			
Strawberries, fresh, frozen or canned (1/2 cup)			W			D			
Blueberries, fresh, frozen or canned (1/2 cup)			W			D			
Peaches, plums or apricots (1 fresh or 1/2 cup canned)			W			D			

VEGETABLES	Never, or less than once per month	1-3 per month	1 per week	2-4 per week	5-6 per week	1 per day	2-3 per day	4-5 per day	6+ per day
Tomatoes (2 slices)			W			D			
Tomato juice or V-8 juice (small glass)			W			D			
Tomato sauce (1/2 cup) e.g., spaghetti sauce			W			D			
Salsa, picante or taco sauce (1/4 cup)			W			D			
Hummus (1/4 cup)			W			D			
String beans (1/2 cup)			W			D			
Beans or lentils, baked, dried (1/2 cup) or soup			W			D			
Tofu, soy burger, soybeans, miso or other soy protein			W			D			
Peas or lima beans (1/2 cup fresh, frz., canned) or soup			W			D			
Broccoli (1/2 cup)			W			D			
Cauliflower (1/2 cup)			W			D			
Cabbage or coleslaw (1/2 cup)			W			D			
Brussels sprouts (1/2 cup)			W			D			
Carrots, raw (1/2 carrot or 2–4 sticks)			W			D			
Carrots, cooked (1/2 cup) or carrot juice (2–3 oz.)			W			D			
Corn (1 ear or 1/2 cup frozen or canned)			W			D			
Mixed or stir fry vegetables (1/2 cup) or soup			W			D			
Yams or sweet potatoes (1/2 cup)			W			D			
Dark orange (winter) squash (1/2 cup)			W			D			
Eggplant, zucchini or other summer squash (1/2 cup)			W			D			
Kale, mustard greens or chard (1/2 cup)			W			D			
Spinach, cooked (1/2 cup)			W			D			
Spinach, raw as in salad (1 cup)			W			D			
Iceberg or head lettuce (1 serving)			W			D			
Romaine or leaf lettuce (1 serving)			W			D			
Peppers: green, yellow or red (2 rings or 1/4 small)			W			D			
Onions as a garnish or in salad (1 slice)			W			D			
Onions as a cooked vegetable or rings (1/2 cup) or soup			W			D			

EGGS, MEAT, ETC.	Never, or less than once per month	1-3 per month	1 per week	2-4 per week	5-6 per week	1 per day	2-3 per day	4-5 per day	6+ per day
Eggs (1): Omega-3 fortified including yolk			W			D			
Eggs (1): Regular eggs including yolk			W			D			
Beef or pork hot dogs (1)			W			D			
Chicken or turkey hot dogs or sausage (1)			W			D			
Chicken/turkey sandwich or frozen dinner			W			D			
Other chicken or turkey, with skin (3 oz.)-including ground			W			D			
Other chicken or turkey, without skin (3 oz.)			W			D			
Bacon (2 slices)			W			D			

© 2021 Wang Y et al. *JAMA Network Open.*

28. ***(continued)*** **For each food listed, fill in the circle indicating how often on average you have used the amount specified during the past year.**

MEAT, FISH	Never, or less than once per month	1–3 per month	1 per week	2–4 per week	5–6 per week	1 per day	2–3 per day	4–5 per day	6+ per day
Salami, bologna, or other processed meat sandwiches			W			D			
Other processed meats, e.g., sausage, kielbasa, etc. (2 oz. or 2 small links)			W			D			
Hamburger (1 patty): Lean or extra lean			W			D			
Hamburger (1 patty): Regular			W			D			
Beef, pork, or lamb as a sandwich or mixed dish, e.g., stew, casserole, lasagna, frozen dinners, etc.			W			D			
Pork as a main dish, e.g., ham or chops (4–6 oz.)			W			D			
Beef or lamb as a main dish, e.g., steak, roast (4–6 oz.)			W			D			
Canned tuna fish (3–4 oz.)			W			D			
Breaded fish cakes, pieces, or fish sticks (1 serving, store bought)			W			D			
Shellfish e.g., shrimp, lobster, scallops, clams as main dish			W			D			
Dark meat fish, e.g., tuna steak, mackerel, salmon, sardines, bluefish, swordfish (3–5 oz.)			W			D			
Other fish, e.g., cod, haddock, halibut (3–5 oz.)			W			D			

BREADS, CEREALS, STARCHES	Never, or less than once per month	1–3 per month	1 per week	2–4 per week	5–6 per week	1 per day	2–3 per day	4–5 per day	6+ per day
Cold breakfast cereal (1 serving)			W			D			
Cooked oatmeal/cooked oat bran (including instant) (1 cup)			W			D			
Other cooked breakfast cereal (1 cup)			W			D			
Bread (1 slice): White bread, including pita			W			D			
Bread (1 slice): Rye/Pumpernickel			W			D			
Bread (1 slice): Whole wheat, oatmeal, other whole grain			W			D			
Crackers (6): Whole grain/whole wheat			W			D			
Crackers (6): Other crackers			W			D			
Bagels, English muffins, or rolls (1)			W			D			
Muffins or biscuits (1)			W			D			
Pancakes or waffles (2 small pieces)			W			D			
Brown rice (1 cup)			W			D			
White rice (1 cup)			W			D			
Pasta, e.g., spaghetti, noodles, couscous. etc. (1 cup)			W			D			
Tortillas: corn or flour (2)			W			D			
French Fries (6 oz. or 1 serving)			W			D			
Potatoes, baked, boiled (1) or mashed (1 cup)			W			D			
Potato chips or corn/tortilla chips (small bag or 1 oz.)			W			D			
Pizza (2 slices)			W			D			

CARBONATED BEVERAGES: Consider the serving size as 1 glass, bottle or can for these carbonated beverages.

BEVERAGES	Never, or less than once per month	1–3 per month	1 per week	2–4 per week	5–6 per week	1 per day	2–3 per day	4–5 per day	6+ per day
CARBONATED BEVERAGES — Low-Calorie (sugar-free) types: Low-calorie beverage with caffeine, e.g., Diet Coke			W			D			
CARBONATED BEVERAGES — Low-Calorie (sugar-free) types: Other low-cal bev. without caffeine, e.g., Diet 7-Up			W			D			
CARBONATED BEVERAGES — Regular types (not sugar-free): Carbonated beverage with caffeine & sugar, e.g., Coke, Pepsi, Mt. Dew, Dr. Pepper			W			D			
CARBONATED BEVERAGES — Regular types (not sugar-free): Other carbonated beverage with sugar, e.g., 7-Up, Root Beer, Ginger Ale, Caffeine-Free Coke			W			D			
OTHER BEVERAGES: Other sugared beverages: Punch, lemonade, sports drinks, or sugared ice tea (1 glass, bottle, can)			W			D			
Beer, regular (1 glass, bottle, can)			W			D			
Light Beer, e.g., Bud Light (1 glass, bottle, can)			W			D			
Red wine (5 oz. glass)			W			D			
White wine (5 oz. glass)			W			D			
Liquor, e.g., vodka, gin, etc. (1 drink or shot)			W			D			
Plain water: bottled, sparkling, or tap (8 oz. cup)			W			D			
Decaffeinated tea, exclude herbal (8 oz. cup)			W			D			
Tea with caffeine (8 oz. cup), including green tea			W			D			
Decaffeinated coffee (8 oz. cup)			W			D			
Coffee with caffeine (8 oz. cup)			W			D			
Dairy coffee drink (hot/cold), e.g., Cappuccino (12 oz.)			W			D			

© 2021 Wang Y et al. *JAMA Network Open.*

28 . *(continued)* **For each food listed, fill in the circle indicating how often on average you have used the amount specified during the past year.**

SWEETS, BAKED GOODS, MISCELLANEOUS	Never, or less than once per month	1–3 per month	1 per week	2–4 per week	5–6 per week	1 per day	2–3 per day	4–5 per day	6+ per day
Milk chocolate (bar or pack), e.g., Hershey's, M&M's									
Dark chocolate, e.g., Hershey's Dark or Dove Dark									
Candy bars, e.g., Snickers, Milky Way, Reese's									
Candy without chocolate (1 oz.)									
Cookies (1) or Brownies (1) – Fat free or reduced fat									
Cookies (1) or Brownies (1) – Other ready made or from mix or dough									
Cookies (1) or Brownies (1) – Home-baked, from scratch									
Doughnuts (1)									
Cake, homemade or ready made (slice)									
Pie, homemade or ready made (slice)									
Jams, jellies, preserves, syrup, or honey (1 Tbs)									
Peanut butter (1 Tbs)									
Popcorn (2–3 cups) – Fat free or light									
Popcorn (2–3 cups) – Regular									
Sweet roll, coffee cake or other pastry (1)									
Snack bars, e.g., Nutrigrain, Kashi, granola, Planter's (1)									
Energy bars or high protein bars, e.g., Clif, Zone, etc.									
Diet nutrition drinks, e.g. Slimfast (1)									
Ensure, Boost or other meal replacement drinks (1)									
Pretzels (1 small bag or serving)									
Peanuts (small packet or 1 oz.)									
Walnuts (1 oz.)									
Other nuts (small packet or 1 oz.)									
Dried cranberries (1/4 cup)									
Mixed dried fruit (1/4 cup)									
Oat bran, other bran (wheat, etc.), added to food (1 Tbs)									
Wheat germ (1 Tbs)									
Chowder or cream soup (1 cup)									
Tomato soup (1 cup)									
Ketchup or red chili sauce (1 Tbs)									
Flaxseed (1 Tbs)									
Garlic, fresh or powdered (1 clove or 4 shakes)									
Olives, any type (3)									
Olive oil added to food or bread (1 Tbs)									
Low-fat or fat-free mayonnaise (1 Tbs)									
Regular mayonnaise (1 Tbs)									
**Salad dressing (1–2 Tbs)** **How often?**									
**Artificial sweeteners (1 packet)** **How often?**									

Salad dressing – Type(s): ◯ Nonfat ◯ Low-fat ◯ Olive oil ◯ Other vegetable oil

Artificial sweeteners – Type(s): ◯ Splenda ◯ Equal ◯ NutraSweet ◯ Sweet'N Low ◯ Saccharin

29. Liver: beef, calf or pork (4 oz.) ◯ Never ◯ Less than 1/mo ◯ 1/mo ◯ 2–3/mo ◯ 1/week or more

Liver: chicken or turkey (1 oz.) ◯ Never ◯ Less than 1/mo ◯ 1/mo ◯ 2–3/mo ◯ 1/week or more

30. How often do you eat fried or sautéed food at home? (Exclude "Pam"-type spray)
◯ Less than once a week ◯ 1–3 times per week ◯ 4–6 times per week ◯ Daily

31. What kind of fat is usually used for frying and sautéing at home? (Exclude "Pam"-type spray)
◯ Real butter ◯ Margarine ◯ Olive oil ◯ Vegetable oil ◯ Veg. shortening ◯ Lard ◯ N/A

32. What kind of fat is usually used for baking COOKIES at home?
◯ Real butter ◯ Margarine ◯ Olive oil ◯ Vegetable oil ◯ Veg. shortening ◯ Lard ◯ N/A

33. What type of cooking oil is usually used at home? (e.g., Mazola Corn Oil) Specify brand and type

34. How often do you eat deep fried chicken, fish, shrimp, clams or onion rings away from home?
◯ Less than once a week ◯ 1–3 times per week ◯ 4–6 times per week ◯ Daily

35. How often do you eat toasted breads, bagel or English muffin (slice or 1 half bagel)?
◯ Less than once a week ◯ 1–3 times per week ◯ 4–6 times per week ◯ Daily ◯ 2+ times/day

Thank you! Please return form to: Nurses' Health Study, 181 Longwood Ave., Boston, MA 02115-5804

Copyright © 2015 President and Fellows of Harvard College. All rights reserved worldwide.

3/8" PERF

© 2021 Wang Y et al. *JAMA Network Open.*

eFigure 2. Study Flow Diagram

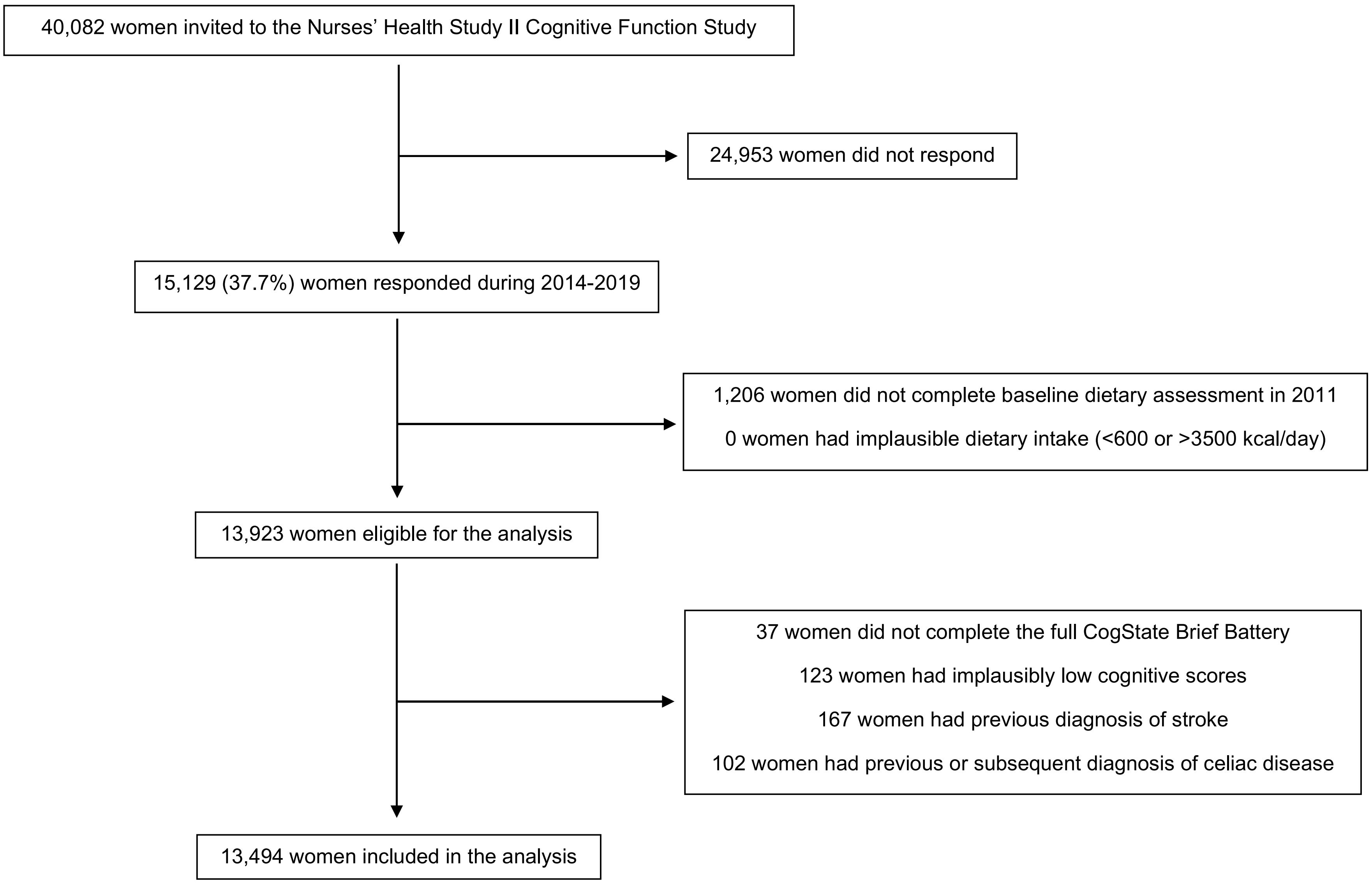

© 2021 Wang Y et al. *JAMA Network Open.*

eReferences

1. Rimm EB, Giovannucci EL, Stampfer MJ, Colditz GA, Litin LB, Willett WC. Reproducibility and validity of an expanded self-administered semiquantitative food frequency questionnaire among male health professionals. *American journal of epidemiology*. 1992;135(10):1114-1126.
2. Willett WC, Sampson L, Stampfer MJ, et al. Reproducibility and validity of a semiquantitative food frequency questionnaire. *American journal of epidemiology*. 1985;122(1):51-65.
3. Lebwohl B, Cao Y, Zong G, et al. Long term gluten consumption in adults without celiac disease and risk of coronary heart disease: prospective cohort study. *bmj*. 2017;357
4. Zong G, Lebwohl B, Hu FB, et al. Gluten intake and risk of type 2 diabetes in three large prospective cohort studies of US men and women. *Diabetologia*. 2018;61(10):2164-2173.
5. Qi J-c, Chen J-x, Wang J-m, Wu F-b, Cao L-p, Zhang G-p. Protein and hordein fraction content in barley seeds as affected by sowing date and their relations to malting quality. *Journal of Zhejiang University Science B*. 2005;6(11):1069.
6. See JA, Kaukinen K, Makharia GK, Gibson PR, Murray JA. Practical insights into gluten-free diets. *Nature reviews Gastroenterology & hepatology*. 2015;12(10):580.
7. Petimar J, Yue Y, Willett W, et al. Validity of Estimated Intake of Flavonoids, Gluten, and the Whole Foods from Which They Are Derived. Poster presented at: Poster presented at: ASN (American Society for Nutrition) 2018; 2018 Jun 10 2018; June 2018; Boston, MA. https://www.eventscribe.com/2018/Nutrition2018/ajaxcalls/PosterInfo.asp?efp=UlhTRFpZVVI0ODYw&PosterID=147034&rnd=0.3035883
8. Salvini S, Hunter DJ, Sampson L, et al. Food-based validation of a dietary questionnaire: the effects of week-to-week variation in food consumption. *International journal of epidemiology*. 1989;18(4):858-867.
9. Willett W, Stampfer MJ. Total energy intake: implications for epidemiologic analyses. *American journal of epidemiology*. 1986;124(1):17.
10. Fredrickson J, Maruff P, Woodward M, et al. Evaluation of the usability of a brief computerized cognitive screening test in older people for epidemiological studies. *Neuroepidemiology*. 2010;34(2):65-75.
11. Sumner JA, Hagan K, Grodstein F, Roberts AL, Harel B, Koenen KC. Posttraumatic stress disorder symptoms and cognitive function in a large cohort of middle-aged women. *Depression and anxiety*. 2017;34(4):356-366.
12. Maruff P, Lim YY, Darby D, et al. Clinical utility of the cogstate brief battery in identifying cognitive impairment in mild cognitive impairment and Alzheimer's disease. *BMC psychology*. 2013;1(1):1-11.
13. Maruff P, Thomas E, Cysique L, et al. Validity of the CogState brief battery: relationship to standardized tests and sensitivity to cognitive impairment in mild traumatic brain injury, schizophrenia, and AIDS dementia complex. *Archives of Clinical Neuropsychology*. 2009;24(2):165-178.
14. Cromer JA, Harel BT, Yu K, et al. Comparison of cognitive performance on the Cogstate brief battery when taken in-clinic, in-group, and unsupervised. *The Clinical Neuropsychologist*. 2015;29(4):542-558.
15. Lim YY, Ellis KA, Harrington K, et al. Use of the CogState Brief Battery in the assessment of Alzheimer's disease related cognitive impairment in the Australian Imaging, Biomarkers and Lifestyle (AIBL) study. *Journal of clinical and experimental neuropsychology*. 2012;34(4):345-358.
16. Fotenos AF, Mintun MA, Snyder AZ, Morris JC, Buckner RL. Brain volume decline in aging: evidence for a relation between socioeconomic status, preclinical Alzheimer disease, and reserve. *Archives of neurology*. 2008;65(1):113-120.
17. Darmon N, Drewnowski A. Does social class predict diet quality? *The American journal of clinical nutrition*. 2008;87(5):1107-1117.
18. Andresen EM, Malmgren JA, Carter WB, Patrick DL. Screening for depression in well older adults: Evaluation of a short form of the CES-D. *American journal of preventive medicine*. 1994;10(2):77-84.
19. Rock P, Roiser J, Riedel W, Blackwell A. Cognitive impairment in depression: a systematic review and meta-analysis. *Psychological medicine*. 2014;44(10):2029.
20. Chiuve SE, Fung TT, Rimm EB, et al. Alternative dietary indices both strongly predict risk of chronic disease. *The Journal of nutrition*. 2012;142(6):1009-1018.
21. Bernstein AM, Rosner BA, Willett WC. Cereal fiber and coronary heart disease: a comparison of modeling approaches for repeated dietary measurements, intermediate outcomes, and long follow-up. *European journal of epidemiology*. 2011;26(11):877-886.

© 2021 Wang Y et al. *JAMA Network Open.*
